# Preclinical–Clinical Discrepancies in Immune Checkpoint Inhibitor Efficacy for Triple-Negative Breast Cancer: Disease Stage, Antibody Properties, Biomarker Alignment, and Model Limitations

**DOI:** 10.3390/cancers18193099

**Published:** 2026-09-23

**Authors:** Yuwei Zhang, Hla Paing, Jing Wang

**Affiliations:** 1 Department of Chemical and Biological Engineering, Iowa State University, Ames, IA 50011, USA; yuwei@iastate.edu; 2 Department of Plant Pathology, Entomology and Microbiology, Iowa State University, Ames, IA 50011, USA; hlampaing1@gmail.com

**Keywords:** immune checkpoint inhibitors, triple-negative breast cancer, pre-clinical models, metastatic disease, Fc-effector, PD-L1

## Abstract

Immune checkpoint inhibitors (ICIs) have emerged as an important class of cancer immunotherapies, yet clinical outcomes do not always align with findings from preclinical studies. This review examines preclinical–clinical discrepancies in ICI efficacy for triple-negative breast cancer (TNBC), with a focus on disease stage, antibody properties, biomarker alignment, and model limitations. Preclinical and clinical findings are most concordant in early-stage TNBC, where ICI-based chemoimmunotherapy improves therapeutic outcomes. In contrast, clinical benefit in metastatic TNBC is more limited and biomarker-dependent, while preclinical studies evaluating ICIs in metastatic TNBC remain scarce, limiting our understanding of immunotherapy resistance at this stage. Currently available TNBC models have not been fully leveraged to represent metastatic disease, in part because their immunosuppressive and metastatic progression has not been systematically characterized. Not all TNBC cell lines develop robust spontaneous metastasis in mouse models, and commonly used experimental metastasis models bypass key stages of primary tumor–immune interaction and metastatic evolution within a systemically immunosuppressive host environment. Additional translational gaps further complicate interpretation, including differences in the immune-effector functions of ICI antibodies used in clinical versus preclinical studies and the lack of standardized approaches to correlate PD-L1 expression in mouse model–derived tumors with clinically defined PD-L1 positivity in patient tumors. Recognizing and addressing these gaps will improve preclinical model selection, strengthen interpretation of ICI efficacy data, and enhance the translational value of preclinical studies for advanced TNBC.

## Simple Summary

Immune checkpoint inhibitor (ICI) efficacy in triple-negative breast cancer (TNBC) varies across disease stages and between preclinical and clinical studies. This review summarizes the evolution of preclinical studies evaluating ICI monotherapy and chemoimmunotherapy in TNBC and examines three underappreciated sources of preclinical–clinical discordance: limitations of metastatic models, differences in antibody isotype and Fc effector function, and inconsistent assessment of PD-L1 expression. Addressing these limitations should improve model selection, strengthen interpretation of therapeutic efficacy, and enhance the translational relevance of preclinical studies in advanced TNBC.

## 1. Introduction

Triple-negative breast cancer (TNBC), defined by the absence of estrogen receptors, progesterone receptors and HER2 expression, is a highly aggressive subtype and accounts for 15–20% of all breast cancer diagnoses [1]. The lack of these therapeutic receptors limits targeted treatment options. Immune checkpoint inhibitors (ICIs) have become important components of TNBC therapy when combined with chemotherapy. ICIs block inhibitory immune pathways that suppress T-cell activation, thereby enhancing antitumor immunity. Although ICI-based immunotherapy has improved outcomes in several cancers, its clinical benefit in TNBC remains modest, particularly in advanced disease, where survival gains are often limited to several months. This highlights the need to improve the magnitude, durability, and patient selectivity of ICI responses in TNBC.

In this review, we summarize and compare clinical and preclinical outcomes of ICI-based monotherapy and chemoimmunotherapy in TNBC, with emphasis on areas of concordance and discrepancy. In early-stage TNBC, preclinical and clinical findings are generally consistent: chemoimmunotherapy is more effective than ICI monotherapy, and neoadjuvant or neoadjuvant–adjuvant treatment provides greater benefit than adjuvant treatment alone. However, most preclinical ICI studies have focused on early-stage primary tumor models, whereas metastatic TNBC models have been less rigorously evaluated. This gap may reflect incomplete characterization of disease progression in commonly used TNBC mouse models, technical difficulty in standardizing metastatic-stage models, or underreporting of negative results from late-stage studies. Regardless of the cause, limited evaluation in metastatic TNBC, together with differences in antibody clones and isotypes, poor biomarker alignment, and insufficient characterization of immunosuppressive and metastatic progression, limits the predictive value of current preclinical models.

## 2. Clinical Trial Outcomes of ICI-Based Chemoimmunotherapy

All three major classes of ICIs—anti-PD-1, anti-PD-L1, and anti-CTLA-4 antibodies—have been evaluated in clinical trials for TNBC (Table 1). The anti-PD-1 antibody pembrolizumab is currently the only FDA-approved ICI for both high-risk early-stage and advanced/metastatic TNBC [2,3]. The anti-PD-L1 antibody atezolizumab previously received accelerated approval with nab-paclitaxel for advanced/metastatic TNBC [4], but this U.S. indication was later voluntarily withdrawn [5]. To date, no anti-CTLA-4 agent has been approved for TNBC.

### 2.1. Anti-PD-1 Antibody

Pembrolizumab and nivolumab are the most extensively studied anti-PD-1 antibodies in TNBC. Pembrolizumab was first evaluated as monotherapy in metastatic TNBC in KEYNOTE-012, KEYNOTE-086, and KEYNOTE-119 [6,7,8,9]. KEYNOTE-012, a phase Ib single-arm cohort in advanced/metastatic PD-L1-positive TNBC, reported an objective response rate (ORR) of 18.5%, providing early evidence that pembrolizumab could induce durable responses in a subset of patients. KEYNOTE-086 showed that first-line PD-L1-positive metastatic TNBC patients had a higher ORR than previously treated patients, yet their median progression-free survival (PFS) remained short. KEYNOTE-119 further showed that pembrolizumab monotherapy did not significantly improve overall survival (OS) compared with physician’s-choice chemotherapy in previously treated metastatic TNBC. These studies indicate that pembrolizumab monotherapy is insufficient for broad clinical efficacy in metastatic TNBC and support the development of chemoimmunotherapy strategies.

I-SPY2, KEYNOTE-173 and KEYNOTE-522 evaluated pembrolizumab-based chemoimmunotherapy in high-risk early-stage TNBC [2,3,10,11,12]. I-SPY2 reported that adding pembrolizumab to neoadjuvant paclitaxel followed by doxorubicin/cyclophosphamide increased the estimated pathologic complete response (pCR) rate in the TNBC subgroup from 22% to 60%. KEYNOTE-173 similarly showed that neoadjuvant pembrolizumab combined with taxane/platinum-based chemotherapy followed by anthracycline/cyclophosphamide achieved an overall pCR rate ~60%. These early-phase results informed KEYNOTE-522, a phase III trial that established the current standard for high-risk early-stage TNBC. In this trial, pembrolizumab plus paclitaxel/carboplatin followed by doxorubicin or epirubicin/cyclophosphamide significantly improved pCR, event-free survival (EFS), and 5-year OS compared with chemotherapy alone. KEYNOTE-522 demonstrated benefit in both PD-L1-positive and negative subgroups. Therefore, the early-stage pembrolizumab indication is not restricted to PD-L1-positive tumors.

For locally recurrent unresectable or metastatic TNBC, pembrolizumab plus chemotherapy is recommended for patients with PD-L1-positive tumors defined by combined positive score (CPS) ≥ 10. KEYNOTE-355 tested pembrolizumab with paclitaxel, nab-paclitaxel, or gemcitabine plus carboplatin and showed significant improvements in median PFS and median OS compared with chemotherapy alone in the CPS ≥ 10 population. However, significant OS benefit was not demonstrated in the CPS ≥ 1 population, supporting the current restriction to CPS ≥ 10 disease [13]. Subsequent studies have tested alternative pembrolizumab combinations. KEYLYNK-009 showed that maintenance pembrolizumab plus olaparib was not superior to pembrolizumab plus continued chemotherapy after induction pembrolizumab/platinum-based chemotherapy [14]. More recently, KEYNOTE-D19/ASCENT-04 showed that pembrolizumab plus sacituzumab govitecan improved PFS compared with pembrolizumab plus standard chemotherapy in first-line PD-L1-positive advanced/metastatic TNBC [15]. Despite these advances, the clinical benefit of pembrolizumab-based therapy in metastatic TNBC remains modest compared with early-stage disease.

Nivolumab has also been evaluated in TNBC but has not led to FDA approval. TONIC tested nivolumab alone or after short induction with radiotherapy or chemotherapy in metastatic TNBC. The overall ORR was 20%, with the highest ORR after doxorubicin induction, suggesting that selected induction therapies may increase sensitivity to PD-1 blockade [16]. OXEL evaluated nivolumab with or without capecitabine in residual high-risk early-stage TNBC after neoadjuvant chemotherapy and surgery. Although nivolumab plus capecitabine increased peripheral immunoscore (PIS) and showed ~4-month longer median invasive disease-free survival (iDFS), differences were not statistically significant [17].

### 2.2. Anti-PD-L1 Antibody

Atezolizumab is the most extensively studied anti-PD-L1 antibody in TNBC. IMpassion130 supported accelerated FDA approval of atezolizumab plus nab-paclitaxel for unresectable locally advanced or metastatic PD-L1-positive TNBC, defined as PD-L1-stained tumor-infiltrating immune cells covering (IC) ≥ 1% of tumor area [4]. In IMpassion130, adding atezolizumab significantly improved PFS in both the intent-to-treat (ITT) population and the PD-L1-positive subgroup. However, the absolute PFS benefit was modest, extending median PFS by 1.7 months in the overall population and 2.5 months in the PD-L1-positive subgroup. The confirmatory IMpassion131 trial failed to improve PFS or OS in either the PD-L1-positive or the overall population, leading to the withdrawal of the U.S. TNBC indication [5]. Subsequent trials further showed inconsistent benefit. IMpassion132 did not improve survival in early-relapsing advanced/metastatic TNBC [18]. In early-stage TNBC, IMpassion031 improved pCR in the neoadjuvant setting, whereas IMpassion030/ALEXANDRA failed to improve 3-year iDFS or OS in the adjuvant setting [19,20]. Thus, although atezolizumab produced an initial signal in PD-L1-positive metastatic TNBC, its overall benefit has been inconsistent.

### 2.3. Anti-CTLA-4 Antibody

Anti-CTLA-4 immunotherapy has been less extensively developed in TNBC than anti-PD-1/PD-L1 therapy. CTLA-4 blockade has limited evidence as monotherapy, and most clinical efforts have focused on dual ICI therapy or bispecific antibodies. In the neoadjuvant BELLINI trial [21], short-course nivolumab with low-dose anti-CTLA-4 ipilimumab produced encouraging chemotherapy-free activity in selected tumor-infiltrating lymphocyte (TIL)-high early-stage TNBC. However, BREASTIMMUNE-03 showed that adjuvant nivolumab plus ipilimumab did not improve DFS compared with capecitabine in high-risk early-stage TNBC with residual disease after neoadjuvant chemotherapy [22]. More recently, bispecific checkpoint blockade has emerged as an alternative strategy. In KN046, an anti-PD-L1 × anti-CTLA-4 bispecific antibody combined with nab-paclitaxel showed clinical activity in first-line locally advanced or metastatic TNBC [23]. Together, these studies suggest that CTLA-4 blockade may have activity in selected TNBC contexts, particularly in combination or bispecific formats, but its clinical benefit remains investigational, and toxicity remains a major limitation.

## 3. Preclinical Outcomes of ICI-Based Chemoimmunotherapy

Preclinical evaluations of ICI-based therapy have focused largely on early-stage TNBC models. Despite species-specific differences in immune biology, tumor heterogeneity, and antibody formats, preclinical and clinical studies support several shared conclusions: chemoimmunotherapy is more effective than ICI monotherapy, chemotherapy choice strongly influences ICI response, neoadjuvant or neoadjuvant–adjuvant strategies outperform adjuvant-only therapy, and dual checkpoint blockade can enhance efficacy in some models. These findings demonstrate the translational value of preclinical models. However, metastatic TNBC remains underexplored preclinically, limiting insight into therapeutic optimization for advanced disease. This section summarizes ICI monotherapy and chemoimmunotherapy across three major classes of TNBC mouse models (Table 2). Because clinical and preclinical studies use different efficacy endpoints, we evaluated concordance based on the durability of therapeutic benefit. In clinical studies, benefit was reflected by improvements in endpoints such as pCR, PFS/EFS, or OS, whereas preclinical benefit was defined by sustained tumor-growth inhibition, delayed progression or metastasis, and/or prolonged survival. Clinical and preclinical findings were considered concordant when both showed a clear therapeutic benefit, limited benefit, or lack of benefit under comparable treatment settings. They were considered discordant when robust tumor control or survival benefit in animal models was not reproduced as meaningful clinical improvement.

### 3.1. Syngeneic Models

Syngeneic TNBC models are generated by implanting murine TNBC-like cell lines into immunocompetent mice, either orthotopically into the mammary fat pad or subcutaneously (Figure 1). These models are widely used because they are rapid, reproducible, cost-effective, and compatible with immune profiling and survival studies. However, these models incompletely recapitulate spontaneous tumor evolution, immune editing, human tumor heterogeneity, and human checkpoint biology. Common syngeneic TNBC-like models include 4T1, EMT6, EO771, and PyMT-derived lines such as AT-3. The 4T1 model is highly aggressive, spontaneously metastatic, and largely refractory to ICIs. 4T1 sublines, including the less metastatic 4T07 and non-metastatic 67NR and the more metastatic 4T1.2, are also used to study TNBC [24,25,26]. Compared to 4T1, EMT6 is less aggressive and less metastatic [27], AT-3 is aggressive, moderately metastatic, and relatively ICI-refractory [28], and EO771 is less aggressiveness, with limited spontaneous metastatic capacity. In contrast, its lung-metastatic derivative, EO771.LMB, shows enhanced metastatic potential [29]. Reported incidence of spontaneous metastasis in untreated 4T1, EMT6, AT3, EO771.LMB and EO771 are 100%, ~60%, ~50%, ~30%, and ~0%, respectively [27,29]. PD-L1 expression also varies across murine TNBC cell lines and primary tumors. In vitro flow cytometric analyses have shown distinct patterns of basal PD-L1 expression across murine mammary tumor cell lines. One study reported the relative order EO771 > 67NR > 4T1.2 ≈ 4T07 > AT-3 ≈ 4T1 [30], whereas another reported EMT6 > EO771 > 4T1 [31]. Direct studies of PD-L1 expression in primary tumors are more limited. One immunohistochemistry (IHC) study showed that PD-L1 expression in primary tumors followed the order 4T1.2 > EO771 > 4T1, and that PD-L1 expression was enhanced after systemic immune stimulation with IFNα or poly(I:C) [30]. Another study measured PD-L1 in whole-tumor lysates by Luminex and reported that PD-L1 levels were highest in 4T1-based tumors, followed by EMT6 and AT-3 [27].

In many prior studies using syngeneic TNBC models to evaluate ICI-based therapies, treatment was initiated when primary tumors reached 50–200 mm^3^ (Table 2), representing an early cancer stage. For comparison, distant metastases in aggressive 4T1 models are detectable by IHC or computed tomography (CT) when primary tumors reach ~1200 mm^3^ [32], whereas metastasis detection in AT3 and EMT6 is more variable and often limited to microscopic lesions or a subset of mice [27,33]. In less aggressive syngeneic TNBC models without consistent lethal metastasis, humane endpoints are often reached when primary tumors reach ~2000 mm^3^. Therefore, most syngeneic ICI studies assess early primary tumor control rather than established metastatic disease. Consistent with clinical observation, preclinical studies also show that ICI monotherapy is often insufficient to control aggressive TNBC models such as 4T1 and AT-3, even when treatment begins early. In contrast, less aggressive models, including EMT6, EO771, and selected PyMT-derived tumors, can show moderate responses, with several studies reporting 50–80% tumor growth inhibition within 2–3 weeks of treatment [34,35,36,37,38,39]. Notably, several preclinical studies also suggest that chemotherapy alone can achieve tumor-suppressive effects comparable to ICI monotherapy in some TNBC models [39,40,41,42].

Combination therapy generally improves outcomes compared with ICI monotherapy. In unresected primary tumor models, these benefits are reflected by stronger inhibition of primary tumor growth, prolonged median survival, and improved event-free survival at defined time points. Beavis et al. reported that AT-3 tumor-bearing mice treated with anti-PD-1 plus anti-CTLA-4 had a median survival of ~50 days, compared with ~30 days after anti-CTLA-4 monotherapy [43]. Bailey et al. reported that a similar dual-ICI strategy in the 4T1 model reduced tumor size by ~82%, whereas anti-CTLA-4 monotherapy reduced tumor size by ~53% on day 14 after tumor inoculation [37]. Orecchioni et al. demonstrated that anti-PD-L1 monotherapy reduced tumor volume from ~900 mm^3^ to ~520 mm^3^ on day 25 after tumor inoculation, while the addition of vinorelbine and cyclophosphamide further reduced tumor volume to ~50 mm^3^ [44]. Kalfeist et al. reported that, in the 4T1 model, median survival increased from 34 days with anti-PD-L1 monotherapy to 45 days with chemoimmunotherapy using anti-PD-L1 plus cisplatin and eribulin. Further addition of anti-TGF-β antibody extended median survival to 62 days and improved survival at the defined study time point [45].

Chemotherapy choice can strongly influence chemoimmunotherapy efficacy [39,40,41,42,44,45,46,47,48,49]. Falvo et al. compared anti-PD-1 therapy combined with different chemotherapeutic agents and found drug-dependent differences in antitumor activity [39]. In 4T1 and EMT6 tumors, anti-PD-1 combined with cyclophosphamide plus vinorelbine showed stronger antitumor activity than combinations with other chemotherapy. Clinical data similarly suggest that the chemotherapy backbone influences chemoimmunotherapy efficacy. For example, the divergent outcomes of IMpassion130 and IMpassion131 suggest that atezolizumab improved outcomes when combined with nab-paclitaxel, but not with paclitaxel [4,5]. Although combination therapies can improve antitumor efficacy, they also increase treatment-associated toxicity compared with monotherapy [50]. However, mouse models have limited ability to predict human immune-related adverse events and clinically relevant toxicities, so toxicity findings from mouse studies should be interpreted cautiously [51].

Post-surgical syngeneic models better approximate clinical treatment settings because they enable evaluation of recurrence, metastasis, and long-term survival after primary tumor removal. In unresected primary tumor models, treatment may reduce metastatic progression, but mice may still require euthanasia when the primary tumor reaches the humane endpoint. Thus, survival often reflects both primary tumor burden and metastatic disease, making it difficult to determine whether therapy specifically controls distant metastasis and improves durable survival. Post-surgical models can better address this limitation, yet they have been rarely used in preclinical ICI studies (Table 2). Existing post-surgical studies suggest that treatment timing and disease stage strongly influence therapeutic benefit. In early-stage models, neoadjuvant chemoimmunotherapy followed by surgery improved long-term survival and suppressed secondary tumor growth. Gao et al. showed that anti-PD-1 plus carboplatin, followed by surgery and secondary tumor inoculation, achieved 150-day survival rates of 90%, 50%, and 70% in the 4T1, EMT6, and EO771 models, respectively, compared with 0% for monotherapy [42]. Liu et al. showed that neoadjuvant plus adjuvant anti-PD-1 therapy outperformed neoadjuvant-only or adjuvant-only therapy in PD-L1-positive 4T1.2 tumors, and dual ICI therapy further improved long-term outcomes, increasing 150-day tumor-free survival to 12%, compared with 6.7% for anti-PD-1 monotherapy [52,53]. In contrast, therapeutic benefit appears more limited in models that have developed metastatic burdens. In a cisplatin-resistant EMT6/CDDP post-surgical metastatic model, Reguera-Nuñez et al. showed that chemoimmunotherapy failed to significantly improve median survival, underscoring the challenge of treating metastatic TNBC [48].

### 3.2. Human TNBC Cell Line–Derived and Patient-Derived Xenograft Models

Cell line-derived xenograft (CDX) models are generated by implanting human TNBC cell lines into immunodeficient mice. Patient-derived xenograft (PDX) models are established by implanting fresh patient tumor fragments or dissociated tumor cells into immunodeficient mice, allowing preservation of patient-specific tumor heterogeneity, histopathology, and therapeutic response [54] (Figure 1). Because ICI-based immunotherapy requires effector cells such as T cells, conventional CDX and PDX models must be combined with human immune-system reconstitution. This is typically achieved by engrafting mice with human peripheral blood mononuclear cells (PBMCs) or human CD34^+^ hematopoietic stem cells (HSCs). Compared with syngeneic models, humanized xenograft models better recapitulate human tumor–immune interactions and provide a more translational platform for evaluating clinical-grade ICIs targeting human immune checkpoints. However, these models remain limited by incomplete human immune reconstitution, and potential graft-versus-host disease, especially in PBMC-humanized models. Their high cost and technical complexity have also limited their use in TNBC ICI studies.

Common human TNBC cell lines used in CDX include MDA-MB-231, MDA-MB-436, and MDA-MB-468. MDA-MB-231 is highly aggressive and metastatic, whereas MDA-MB-436 and MDA-MB-468 exhibit lower metastatic potential, although all three can consistently develop distant metastases [55,56]. These models also differ in PD-L1 expression. MDA-MB-231 is considered PD-L1-high, MDA-MB-436 relatively PD-L1-low, and MDA-MB-468 PD-L1-negative [57,58,59]. In CD34^+^ HSC-humanized mouse models bearing MDA-MB-231 tumors, pembrolizumab and nivolumab have shown 20–50% tumor growth inhibition [60,61,62,63]. Atezolizumab has also shown efficacy [63], whereas ipilimumab has generally shown limited or no antitumor efficacy [62]. These findings differ from some syngeneic models, where anti-CTLA-4 can be more active, potentially reflecting species-specific checkpoint biology, antibody isotype, and Fcγ receptor engagement. Notably, although combining ICIs with chemotherapy can further reduce MDA-MB-231 tumor burden in some studies, the added benefit may be comparable to that achieved by chemotherapy alone [61,64]. Similarly, studies in humanized MDA-MB-231 xenograft models suggest that dual ICI combinations, as well as combinations of ICIs with anti-TGF-β, anti-TGF-βRII, or immunostimulatory agonist antibodies such as anti-CD137, do not consistently outperform ICI monotherapy [62,63].

PDX models enable correlation of ICI responses with clinically relevant PD-L1 expression patterns. Rosato et al. evaluated anti-PD-1 therapy in CD34^+^ HSC-humanized NSG mice bearing TNBC PDX tumors and showed that pembrolizumab or nivolumab reduced tumor growth and improved survival in PD-L1-positive PDX models but not in PD-L1-negative models. In contrast, anti-CTLA-4 monotherapy did not provide therapeutic benefit. These findings support the use of humanized TNBC PDX models to study differential sensitivity and resistance to ICI-based therapy [65]. Humanized xenograft studies have also demonstrated that anti-PD-1 treatment can increase human CD8^+^ IFN-γ^+^ T-cell responses, and that combination regimens can produce greater tumor inhibition than monotherapy, supporting their use for prioritizing rational ICI combinations [60]. When multiple PDX models representing different molecular and immune phenotypes are evaluated in parallel, these systems can therefore help prioritize therapeutic combinations, identify responsive tumor subsets, and guide biomarker development before clinical testing.

Though humanized CDX and PDX models provide an important advantage over conventional xenografts by permitting evaluation of human tumor–immune interactions and responses to human-specific immunotherapies, several limitations should be considered when interpreting results from these systems. In addition to incomplete immune reconstitution and the risk of graft-versus-host disease, incompatibility between the HLA background of the implanted tumor and that of the immune-cell donor can generate alloreactive responses that do not reflect physiologic tumor-specific immunity and may confound assessment of ICI efficacy [66,67,68]. Ideally, tumor and immune cells should be autologous or appropriately HLA matched, although this is often difficult to achieve in practice. Moreover, current humanized mouse platforms incompletely reproduce the full diversity and function of the human immune system. Depending on the humanization strategy, myeloid cells, NK cells, mature B cells, dendritic cells, lymphoid structures, and antigen-specific humoral responses can be poorly developed or functionally impaired because of species-specific differences in cytokines and other developmental signals. Donor-to-donor variability in immune reconstitution further contributes to experimental heterogeneity [69,70,71]. Thus, although humanized CDX and PDX models more closely approximate human tumor–immune interactions than conventional xenografts, they remain partially reconstituted chimeric systems rather than complete representations of human antitumor immunity.

### 3.3. Genetically Engineered Mouse Models

Genetically engineered mouse models (GEMMs) are valuable because tumors arise spontaneously in the native mammary gland, develop through progressive stages of tumorigenesis, and interact continuously with an intact immune system and native stromal microenvironment (Figure 1). These features allow investigation of immune surveillance, immune editing, metastatic progression, and treatment response in a setting that can more closely reproduce the co-evolution of tumor and host immunity than transplantation models. GEMMs are also useful for testing how defined oncogenic drivers or tumor-suppressor alterations shape the immune microenvironment and influence sensitivity or resistance to immunotherapy.

Several GEMMs reproduce basal-like or TNBC-associated biology. The C3(1)-SV40 T-antigen model develops highly penetrant basal-like mammary tumors, while BRCA1-deficient models, particularly those combining Brca1 and Trp53 loss, generate high-grade tumors relevant to BRCA1-associated TNBC. Additional Trp53- or Rb1-based models can produce basal-like or claudin-low phenotypes [72,73,74,75]. These models provide complementary platforms for studying how distinct TNBC-associated genetic programs influence tumor–immune interactions and responses to immune checkpoint blockade. However, they have not been widely used for systematic evaluation of immunotherapy efficacy because GEMM studies are generally more costly and time-consuming than transplantation models.

The MMTV-PyMT model is among the most widely used mammary tumor GEMMs for studying immunotherapy efficacy. In this model, polyoma middle T antigen is expressed under the MMTV promoter, driving multifocal mammary tumor development. Female MMTV-PyMT mice on the FVB/N and C57BL/6 backgrounds develop palpable mammary tumors with reported mean latencies of approximately 6–7 weeks and 10–11 weeks of age, respectively, and tumors commonly metastasize to the lung at later stages [76,77]. Although MMTV-PyMT tumors are predominantly luminal-like rather than TNBC, this model remains informative for studying ICI response because it develops spontaneous mammary tumors within an intact immune system and exhibits a strongly immunosuppressive, myeloid-rich tumor microenvironment. PyMT tumors generally show limited responses to ICI monotherapy, while their sensitivity can be altered by chemotherapy or other immune-modulating interventions, making the model useful for investigating mechanisms of ICI resistance and strategies that restore antitumor immunity.

MMTV-PyMT tumors generally show low PD-L1 expression on tumor cells, although PD-L1 can be detected in the tumor microenvironment [49,78]. ICI responses in MMTV-PyMT models have been limited. In FVB/N mice, Redmond et al. and Lal et al. showed that early anti-PD-L1, anti-CTLA4, or combination therapy failed to suppress tumor development or improve survival [79,80]. In C57BL/6 mice, Sirait-Fischer et al. reported that anti-PD-1 monotherapy and anti-PD-1 plus doxorubicin reduced tumor burden by 40–50% when treatment was initiated at an early cancer stage [78]. In B6.FVB mice, Kalfeist et al. showed that combined anti-PD-L1 and anti-TGF-β therapy alone did not provide therapeutic benefit, but when combined with cisplatin-eribulin chemotherapy, it extended median survival from ~33 to ~80 days and greatly reduced tumor lesions in treated mice, although long-term survival remained limited [45].

Overall, currently available syngeneic, CDX, PDX, and GEMM models provide valuable platforms for evaluating ICI-based therapies in TNBC and generating insights that may inform clinical trials. However, each model has distinct advantages and limitations. To better interpret preclinical findings and correlate them with clinical outcomes, it is important to understand the factors that contribute to discrepancies between preclinical and clinical data.

## 4. Gaps Between Clinical Trials and Pre-Clinical Evaluation

### 4.1. Antibody Isotype and Fcγ Receptor Engagement

Antibody isotype is an important but overlooked difference between clinical and preclinical ICI studies. Clinical ICIs are typically human or humanized IgG1 or IgG4 antibodies, which differ in Fc-mediated effector functions [81]. IgG1 has stronger Fcγ receptor (FcγR) engagement and complement activation than IgG4, which can convert an antibody from a primarily blocking agent into an immune-effector molecule capable of mediating antibody-dependent cellular cytotoxicity or phagocytosis (ADCC/ADCP) and depletion of target-expressing cells [82]. Therefore, antibody isotype and Fc-effector function can strongly influence whether an ICI acts mainly through checkpoint blockade or also through Fc-dependent immune-cell modulation.

The Fc design of clinical ICIs differs by checkpoint target. Clinical anti-PD-1 antibodies are generally IgG4 with minimized FcγR engagement, a design intended to block PD-1 signaling while avoiding depletion of PD-1^+^ activated T cells. In contrast, clinical anti-PD-L1 antibodies are often IgG1, although many are Fc-engineered to reduce unwanted depletion of PD-L1^+^ immune cells. FcγR engagement by anti-PD-L1 antibodies may help eliminate PD-L1^+^ tumor cells or suppressive myeloid cells, but it may also deplete beneficial antigen-presenting cells or promote unwanted inflammation [83]. Clinical anti-CTLA-4 antibodies are generally IgG1, which support Fc-mediated depletion of CTLA-4^+^ regulatory T cells in the tumor microenvironment [84].

In mouse studies, ICI antibodies are commonly non-mouse anti-mouse antibodies with different species origins and isotypes. Examples include anti-PD-1 clones RMP1-14 and 4H2 as rat IgG2a, anti-PD-L1 clones 10F.9G2 and B7-H1 as rat IgG2b, and anti-CTLA-4 clone 9H10 as hamster IgG or clone 9D9 as mouse IgG2b [85]. Mouse and rat IgG subclasses differ in FcγR engagement, with IgG2a exhibiting the strongest FcγR binding, followed by IgG2b, whereas IgG1 has weaker FcγR engagement [86]. This creates a major translational mismatch. For example, clinical anti-PD-1 antibodies are generally Fc-attenuated human IgG4, whereas commonly used preclinical anti-mouse PD-1 antibodies are often rat IgG2a, which may have greater FcγR engagement and could therefore influence antitumor activity through Fc-dependent mechanisms [85]. These differences in antibody species, clone, and isotype complicate direct comparison between preclinical and clinical outcomes.

Mechanistic studies have demonstrated that FcγR engagement can substantially influence ICI efficacy. In colon cancer models, Dahan et al. showed that FcγR binding compromises anti-PD-1 activity but enhances anti-PD-L1 activity, while Ingram et al. demonstrated that CTLA-4 blockade requires an Fc domain capable of engaging FcγR for optimal antitumor efficacy [87,88]. Consistent findings have also been reported in TNBC models (Table 2). Schofield et al. reported that, in the EMT6 model, the survival rate at day 90 after tumor inoculation increased from 0% in control-treated mice to 55% and 90% after anti-CTLA-4 mIgG1 and mIgG2b monotherapy, respectively, likely reflecting stronger FcγR engagement and Treg depletion by mIgG2b [38]. In contrast, strong FcγR engagement can be detrimental for anti-PD-1 therapy. Anti-PD-1 IgG2a showed lower efficacy than anti-CTLA-4 IgG2b in EO771 and PyMT-derived syngeneic mammary tumor models [36,89], whereas clinical pembrolizumab (anti-PD-1 IgG4) showed greater efficacy than ipilimumab (anti-CTLA-4 IgG1) in hu-NSG PDX models [65].

Together, these findings highlight an important translational gap: the efficacy ranking of ICIs in syngeneic mouse models may be shaped by antibody isotype and Fc-effector function rather than by checkpoint target alone. Future preclinical studies should use antibody formats that better mimic clinical ICIs, including Fc-silent or Fc-attenuated anti-PD-1 antibodies and Fc-competent anti-CTLA-4 antibodies when Treg depletion is intended.

### 4.2. Biomarker Alignment of TNBC Tumors

Biomarker alignment between mouse model-derived murine or human TNBC tumors and patient tumors is another factor affecting translational interpretation. Key biomarkers include PD-L1 expression and scoring, TILs, tumor mutational burden, antigen-presentation capacity, and immune-suppressive myeloid signatures [90]. In clinical TNBC, PD-L1 positivity for pembrolizumab-based therapy is commonly defined using the PD-L1 IHC 22C3 pharmDx assay with a CPS ≥ 10. CPS is calculated as the number of PD-L1-staining tumor cells, lymphocytes, and macrophages divided by the total number of viable tumor cells and multiplied by 100. In atezolizumab-based TNBC trials, PD-L1 positivity was defined using the VENTANA SP142 IHC assay as PD-L1-positive tumor-infiltrating immune cells occupying ≥ 1% of the tumor area, reported as IC ≥ 1% [91]. In both assays, tumor and immune cells are distinguished by tissue morphology. These standardized clinical assays differ from many preclinical PD-L1 IHC analyses, which often use semi-quantitative staining intensity, percent-positive cells, or H-scores and may not distinguish tumor-cell from immune-cell PD-L1 [92].

Additionally, only limited studies have characterized PD-L1 expression and related immune biomarkers in commonly used TNBC cell lines and their primary tumors [27,30,31,49,56,57,78,93,94,95]. No standardized framework currently exists for relating PD-L1 expression in syngeneic and CDX tumors to clinically defined PD-L1 positivity in patient tumors. As a result, preclinical PD-L1 IHC findings cannot be directly equated with clinical PD-L1 positivity. PDX models may partially address this limitation by preserving patient-derived tumor architecture and biomarker patterns, but the high cost, technical complexity, and variable engraftment efficiency of these models limit their widespread use.

To improve the translational relevance of preclinical TNBC models, tumors generated from humanized CDX models should be systematically characterized for PD-L1 expression using clinically relevant assays. Similarly, tumors generated from syngeneic models should be evaluated using standardized PD-L1 staining and scoring approaches, with clear distinction between tumor-cell and immune-cell PD-L1 expression. Other clinically relevant features, such as TIL abundance, CD8^+^ T-cell localization, myeloid-cell infiltration, and tumor mutational burden, should also be systematically assessed in these mouse model–derived tumors. Integrating these biomarker data could better align preclinical model selection with clinically relevant TNBC immune phenotypes and improve the predictive value of preclinical ICI studies. However, species-appropriate reagents and murine scoring systems should be carefully validated and calibrated against clinically used human criteria, such as CPS and IC scoring.

### 4.3. Metastatic TNBC Preclinical Models

Preclinical evaluation of ICIs in metastatic TNBC remains limited, restricting understanding of immunotherapy resistance mechanisms and the development of optimized treatment strategies for metastatic disease. Clinically, metastatic TNBC is defined by the spread of tumor cells to distant organs or distant lymph nodes, which is commonly detected using imaging modalities such as CT or biopsy-based confirmation. At the metastatic stage, tumor progression is often accompanied by systemic immunosuppression, a state in which immune cells are unable to effectively recognize or eliminate tumor cells [96]. This immunosuppressive state is mediated by multiple mechanisms, and it is both a consequence of cancer progression and a driver of metastatic outgrowth and resistance to immunotherapy [97,98]. An ideal metastatic TNBC preclinical model should reproduce not only detectable distant metastatic lesions, but also the immune alterations associated with advanced disease. Systemic immunosuppression is an important characteristic of late-stage disease and can be validated by ex vivo assays demonstrating myeloid-cell-mediated suppression of T-cell proliferation [99]. Because metastatic immune states are heterogeneous across models and metastatic sites, features other than systemic immunosuppression may also indicate advanced disease, including the development of organ-specific immunosuppressive niches and alterations in the abundance or functional state of myeloid and lymphoid populations [100,101,102,103]. These immune alterations may evolve with increasing metastatic burden and disease progression. Thus, systemic immunosuppression should be considered an important indicator of advanced metastatic disease, while complementary immune features may provide additional evidence of late-stage progression in specific models. However, currently available implantation-based mouse models have several limitations for modeling immune status in metastatic TNBC.

In implantation models, primary tumors often grow rapidly, whereas spontaneous metastasis and systemic immunosuppression require time to develop through tumor-immune interactions. In the highly metastatic 4T1 model, systemic immunosuppression is typically evident when primary tumors reach >600 mm^3^ [104,105], and distant metastatic lesions become detectable by CT or IHC when primary tumors reach ~1200 mm^3^ [32]. Similarly, previous studies using other breast cancer models, including MVT1, EMT6, AT3, 66CL4, and MMTV-PyMT, have shown that macroscopic or imaging-detectable metastases generally emerge when primary tumors approach or exceed ~1000 mm^3^ [27]. Notably, precise stage definitions remain model-dependent and have not been systematically established across preclinical breast cancer models. If therapy begins after metastases are detectable, the treatment window before primary tumors reach humane endpoint is short, making it difficult to evaluate therapeutic efficacy against established metastases. Post-surgical models can address this limitation by resecting primary tumors and initiating treatment after metastases emerge, thereby more closely resembling clinical metastatic relapse. However, this approach also has important constraints. Several TNBC cell lines, including EMT6, AT3, and EO771, exhibit limited or inconsistent spontaneous distant metastasis. Moreover, when primary tumors are resected early, systemic immunosuppression and the associated metastatic niche may not yet be fully established, potentially reducing the extent to which these models reproduce the immune context of advanced metastatic disease. Although we found that systemic immunosuppression and metastatic growth in the 4T1 model can continue to develop after early primary tumor resection, such as at 300–400 mm^3^ [105], this process remains poorly characterized in other TNBC models. Another underestimated issue is cross-laboratory variability. The aggressiveness and metastatic behavior of cancer cell lines can vary because of differences in cell preservation, passage number, culture conditions, metabolic state, and clonal drift. Such variability may contribute to conflicting immunotherapy results. Standardized characterization of tumor growth, metastatic potential, and host immune status at treatment initiation would improve reproducibility and cross-study comparison.

Experimental metastasis models are often used to address the low or variable spontaneous metastasis [106]. In these models, single-cell suspensions of cancer cells are directly introduced into the circulation by intravenous or intracardiac injection, allowing rapid seeding of distant organs. Organ-specific metastasis can also be modeled by direct injection into specific sites, such as intraosseous injection for bone metastasis or intraportal-vein approaches for liver metastasis [107,108,109]. These models are useful for studying tumor-cell colonization and organ-specific outgrowth, but they bypass several key steps of the metastatic cascade, including primary tumor formation, local invasion, intravasation, immune editing, and spontaneous dissemination. Because experimental metastasis models are often established in tumor-free, healthy mice, they may not recapitulate the systemic immunosuppressive environment generated by progressive primary tumors. Consequently, tumor cells introduced directly into distant organs may either be eliminated by intact immune surveillance or grow under selective pressures that differ from those operating during spontaneous metastatic dissemination [105]. Even when these seeded cells form metastatic lesions, the resulting tumors may differ biologically from spontaneous metastases, and the organ-specific immune microenvironment may not reflect the niche shaped by primary tumor-driven immune editing. A potential improvement is to combine orthotopic primary tumor development with experimental metastasis. In this approach, TNBC cells are first implanted into the mammary fat pad to allow primary tumor growth and the development of systemic immunosuppression. After the primary tumor is resected at a defined immunosuppressive stage, cancer cells are then introduced intravenously or through another route to accelerate metastatic colonization. This design may better model metastatic outgrowth in an immunosuppressed host. To establish this type of model, it is necessary to define when systemic immunosuppression emerges in each TNBC preclinical model. At present, this information is only characterized in the 4T1 model and remains limited for many other syngeneic TNBC models. More systematic characterization of tumor growth kinetics, metastatic timing, immune suppression, and myeloid-T-cell interactions across available TNBC models will improve the establishment of metastatic TNBC models for ICI evaluation.

Compared with implantation models, GEMMs can better mimic spontaneous tumor development because they preserve the co-evolution of tumor cells, stromal cells, vasculature, and immune populations during cancer progression [110]. These features make GEMMs valuable for studying stage-dependent responses to ICI-based therapy in an immunocompetent setting. However, GEMMs also have limitations, including long and variable tumor latency, asynchronous multifocal tumor development, high cost, breeding complexity, murine rather than human checkpoint biology, and incomplete representation of human TNBC heterogeneity. In addition, many GEMMs develop multiple primary mammary tumors, which complicates surgical resection and limits their use for modeling post-surgical metastatic relapse. Similar to implantation models, the timing and mechanisms of systemic immunosuppression in GEMMs remain poorly characterized.

### 4.4. Tumor Microenvironment Heterogeneity

The clinical behavior of TNBC is strongly influenced by the tumor microenvironment (TME), which includes immune and stromal cell populations as well as local metabolic conditions such as hypoxia, nutrient availability, extracellular acidity, and lactate accumulation. At the cellular level, the abundance, spatial organization, and functional state of tumor-infiltrating lymphocytes, macrophages, myeloid-derived suppressor cells (MDSCs), neutrophils, fibroblasts, and other stromal populations can profoundly influence tumor progression and immunotherapeutic response. In patients with TNBC, higher stromal TIL levels are consistently associated with improved prognosis and a greater likelihood of pathological complete response to neoadjuvant therapy, whereas enrichment of immunosuppressive myeloid and stromal populations is generally associated with less favorable immune states and poorer outcomes [111,112]. These differences are also reflected in clinically relevant TNBC molecular subclasses. For example, basal-like immune-activated and immunomodulatory tumors exhibit stronger immune-response signatures and generally more favorable outcomes, whereas basal-like immune-suppressed, mesenchymal, and luminal androgen receptor tumors display distinct immune, stromal, and metabolic phenotypes [113].

Similar heterogeneity is observed among murine breast cancer models. Commonly used syngeneic tumors, including 4T1, EMT6, EO771, AT3, and 66CL4, differ substantially in the abundance and activation state of T cells, NK cells, macrophages, neutrophils, and other myeloid populations, as well as in their baseline responsiveness to immune checkpoint blockade. Comparative profiling studies have identified models ranging from relatively T-cell-inflamed tumors to those dominated by suppressive myeloid populations or characterized by weak lymphocyte infiltration [27,103]. For example, 4T1 tumors induce pronounced systemic and intratumoral expansion of MDSCs, particularly granulocytic MDSCs, which can suppress T-cell proliferation and contribute to an immunosuppressive host state [114,115]. In contrast, models such as EMT6 can exhibit greater baseline lymphocytic infiltration and stronger sensitivity to PD-1/PD-L1 pathway blockade under selected experimental conditions [116,117]. Thus, even models commonly used to represent the same clinical breast cancer subtype can exhibit markedly different immune states.

The metabolic TME provides an additional layer of heterogeneity. Rapid tumor growth can generate hypoxia, glucose depletion, extracellular acidification, and lactate accumulation, which can suppress cytotoxic T-cell and NK-cell activity while favoring regulatory T cells and immunosuppressive myeloid phenotypes [118,119,120]. Murine breast cancer studies have similarly demonstrated substantial differences in glycolytic activity, oxidative phosphorylation, lipid metabolism, hypoxia-associated signaling, and inflammatory pathways across models [27,121]. Manipulation of lactate metabolism, hypoxia signaling, or nutrient availability can alter immune-cell function and, in some settings, increase responsiveness to immunotherapy [119]. Cellular and metabolic features of the TME are therefore tightly interconnected: tumor-derived metabolites regulate immune-cell recruitment and function, while immune and stromal populations can reshape nutrient availability, extracellular matrix organization, cytokine signaling, and local oxygenation. Consequently, tumors with similar growth kinetics or histological appearance may nevertheless exist within substantially different immune and metabolic environments [122,123].

Despite this diversity among experimental models, the range of TMEs represented by currently available murine tumors remains substantially narrower than the heterogeneity observed in patients. Human breast cancers arise in genetically, environmentally, and clinically diverse individuals and exhibit extensive interpatient, intratumoral, spatial, and temporal heterogeneity. In contrast, syngeneic studies generally rely on a relatively small number of established tumor cell lines implanted into genetically homogeneous inbred mouse strains under standardized laboratory conditions. Thus, heterogeneity among available murine models should not be assumed to capture the full breadth of immune, stromal, and metabolic states observed in clinical breast cancer.

Translation is further complicated by fundamental differences between murine and human immunity. Although syngeneic models preserve an intact immune system and species-matched tumor–host interactions, mice and humans differ in immune-cell development and composition, cytokine and chemokine networks, Fc-receptor biology, antigen presentation, myeloid-cell phenotypes, and the expression and function of several therapeutic immune targets [124,125]. These species-specific differences can influence both baseline tumor immunity and responses to immune checkpoint blockade, such that apparently similar immune phenotypes in mouse and human tumors may not be functionally equivalent [126,127].

Another important limitation is the uneven representation of breast cancer subtypes among available immunocompetent models. Most widely used syngeneic breast cancer models exhibit triple-negative or basal-like characteristics, whereas robust hormone receptor-positive and HER2-positive syngeneic models are comparatively scarce [128,129,130,131]. This imbalance limits systematic evaluation of immunotherapy across clinically defined breast cancer subclasses and makes it difficult to determine whether differences in ICI sensitivity among human breast cancer subtypes arise primarily from tumor-cell-intrinsic programs, the surrounding TME, or interactions between the two.

Progress has nevertheless been made in characterizing individual murine breast cancer models with respect to immune-cell composition, inflammatory signaling, checkpoint expression, hypoxia-associated pathways, metabolic programs, and sensitivity to immunotherapy [117,132]. Recent studies have also begun to compare multiple models systematically [27,124,133]. However, a comprehensive framework that integrates these cellular and metabolic TME features across commonly used breast cancer models and quantitatively aligns them with corresponding clinical breast cancer phenotypes remains lacking. Matching a preclinical model to a clinical subtype solely on the basis of tumor-cell molecular characteristics may therefore be insufficient. Future studies integrating TIL density and spatial organization, myeloid and stromal composition, checkpoint expression, hypoxia, glycolytic activity, lactate accumulation, and other metabolic features with patient-derived multi-omic and spatial datasets could establish model-to-patient TME correspondence maps [134,135,136,137]. Such a TME-informed framework could improve preclinical model selection, facilitate interpretation of discrepant ICI results across models, and ultimately enhance the translational predictive value of breast cancer immunotherapy studies.

## 5. Conclusions

Overall, this review summarizes and compares the clinical and preclinical outcomes of ICI-based therapies for TNBC and discusses areas of consistency and discrepancy between these settings. Although developing new TNBC preclinical models for ICI evaluation is important, it is often costly and time-consuming. In the near term, addressing limitations in currently available models may provide a faster and more efficient path toward generating translational insights. Key optimization strategies include using ICI antibody formats that more closely resemble clinically used antibodies, developing standardized PD-L1 scoring systems for mouse model–derived tumors to improve alignment with clinical tumor characterization, systematically defining immunosuppressive and metastatic progression in each available TNBC model, and integrating orthotopic and experimental metastasis approaches to establish more representative metastatic models. Ultimately, closer alignment between preclinical model characteristics and clinical TNBC phenotypes should improve the predictive value of preclinical studies and support more rational design of future immunotherapy trials. Well-characterized preclinical models can help prioritize therapeutic combinations, identify biomarkers of response or resistance, and guide patient selection and treatment sequencing before clinical testing. Conversely, emerging clinical data can be used to refine model selection and experimental design, creating a more iterative and clinically informed translational framework.

**Table 1:** Clinical trial outcomes of ICI-based therapy for TNBC.

Trial Name and ID	Therapy Groups	Start Year	Phase	Disease Setting	Line of Therapy	Outcomes	FDA Approval	Refs.
**Anti-PD-1: pembrolizumab**								
KEYNOTE-012 (NCT01848834)	Single arm: pembrolizumab (10 mg/kg IV every 2 weeks); *n* = 27 evaluable.	2013	Ib	Advanced/metastatic PD-L1-positive TNBC; treatment-naïve and pretreated patients	Mixed; many patients were previously treated	ORR: 18.5%. Median PFS: 1.9 months. Median OS: 11.2 months.	/	Nanda et al. 2016 [6]
I-SPY2 pembrolizumab arm (NCT01042379)	Arm 1: pembrolizumab (200 mg IV every 3 weeks for 4 cycles) + weekly paclitaxel followed by doxorubicin/cyclophosphamide; *n* = 69 with HER2-negative breast cancer, including *n* = 29 TNBC. Arm 2: standard neoadjuvant chemotherapy control; *n* = 181.	2014	II	High-risk stage II/III HER2-negative breast cancer, including TNBC	First-line neoadjuvant treatment	Arm 1 (TNBC subgroup) vs. Arm 2: pCR: 60% vs. 22%.	/	Nanda et al. 2020 [10]
ENHANCE-1 (NCT02513472)	Single arm: pembrolizumab (200 mg IV every 3 weeks, for up to 2 years) + eribulin mesylate; *n* = 167.	2015	Ib/II	Metastatic TNBC	First-line to third-line metastatic treatment	ORR: 23.4% overall, 25.8% in first-line patients, and 21.8% in later-line patients.		Tolaney, et al. 2021 [138]
KEYNOTE-086 Cohort A (NCT02447003)	Single arm: pembrolizumab (200 mg IV every 3 weeks, for up to 2 years); *n* = 170.	2015	II	Previously treated metastatic TNBC	Second-line or later-line metastatic treatment	ORR: 5.3% overall. Median PFS: 2.0 months. Median OS: 9.0 months.	/	Adams, et al. 2019 [8]
KEYNOTE-086 Cohort B (NCT02447003)	Single arm: pembrolizumab (200 mg IV every 3 weeks, for up to 2 years); *n* = 84.	2015	II	Previously untreated metastatic PD-L1-positive TNBC	First-line metastatic treatment	ORR: 21.4%. Median PFS: 2.1 months. Median OS: 18.0 months.	/	Adams, et al. 2019 [7]
KEYNOTE-119 (NCT02555657)	Arm 1: pembrolizumab (200 mg IV every 3 weeks, for up to 2 years); *n* = 312. Arm 2: investigator’s-choice single-agent chemotherapy; *n* = 310. Chemotherapy included capecitabine, eribulin, gemcitabine, or vinorelbine.	2015	III	Previously treated metastatic TNBC	Second- or third-line metastatic treatment	Arm1 vs. Arm 2: Median OS, CPS ≥ 10: 12.7 vs. 11.6 months (HR = 0.78, *p* = 0.057). Median OS, CPS ≥ 1: 10.7 vs. 10.2 months (HR = 0.86, *p* = 0.073). Median OS, ITT: 9.9 vs. 10.8 months (HR = 0.97, *p* = 0.82). Median PFS, ITT: 2.1 vs. 3.3 months (HR = 1.60, *p* value not reported).	/	Winer, et al. 2021 [9]
KEYNOTE-173 (NCT02622074)	Single arm: pembrolizumab (200 mg IV every 3 weeks, for up to 9 cycles) + neoadjuvant chemotherapy; *n* = 60. Chemotherapy options included taxane/platinum-based therapy followed by doxorubicin or epirubicin + cyclophosphamide.	2016	Ib	High-risk early-stage nonmetastatic TNBC	First-line neoadjuvant treatment	pCR: ~60% overall.	/	Schmid, et al. 2020 [11]
KEYNOTE-162/TOPACIO (NCT02657889)	Single arm: pembrolizumab (200 mg IV every 3 weeks) + niraparib (a PARP inhibitor); *n* = 55 TNBC.	2016	I/II	Advanced or metastatic TNBC	Later-line metastatic treatment	ORR: ~21% in the TNBC subgroup. DCR: ~49–65%.	/	Vinayak, et al. 2019 [139]
IMPRIME 1 (NCT02981303)	Single arm: pembrolizumab (200 mg IV on day 1 of 21-day cycle) + Imprime PGG (an immunomodulator); *n* = 42.	2016	II	Previously treated metastatic TNBC	After progression beyond first-line therapy	ORR: 14.3% (1 CR + 5 PR). Median PFS: 2.7 months. Median OS: 16.4 months.	/	Stopeck, et al. 2025 [140]
KEYNOTE-355 (NCT02819518)	Arm 1: pembrolizumab (200 mg IV every 3 weeks) + chemotherapy; *n* = 566. Arm 2: placebo + chemotherapy; *n* = 281. Chemotherapy options included paclitaxel, nab-paclitaxel, or gemcitabine + carboplatin.	2017	III	Previously untreated locally recurrent inoperable or metastatic TNBC	First-line treatment for advanced/metastatic disease	Arm 1 vs. Arm 2: Median PFS, CPS ≥ 10: 9.7 vs. 5.6 months (HR = 0.65, *p* = 0.0012). Median OS, CPS ≥ 10: 23.0 vs. 16.1 months (HR = 0.73, *p* = 0.0185). Median OS, CPS ≥ 1: 17.6 vs. 16.0 months (HR = 0.86, *p* = 0.1125). Median OS, ITT: 17.2 vs. 15.5 months (HR = 0.89).	FDA approval in 2020	Cortes, et al. 2022 [13]
KEYNOTE-522 (NCT03036488)	Arm 1: pembrolizumab + neoadjuvant chemotherapy, followed by adjuvant pembrolizumab; *n* = 784. Pembrolizumab was given 200 mg IV every 3 weeks, including 8 cycles during neoadjuvant therapy and 9 cycles during adjuvant therapy. Arm 2: placebo + neoadjuvant chemotherapy, followed by adjuvant placebo; *n* = 390. Neoadjuvant chemotherapy options included paclitaxel + carboplatin, followed by doxorubicin or epirubicin + cyclophosphamide.	2017	III	High-risk early-stage stage II–III TNBC	First-line neoadjuvant and adjuvant treatment	Arm 1 vs. Arm 2, all patients, regardless of PD-L1 status: pCR: 64.8% vs. 51.2% (*p* < 0.001). EFS, 3-year: 84.5% vs. 76.8% (HR = 0.63, *p* < 0.001). 5-year OS: 86.6% vs. 81.7% (HR = 0.66, *p* = 0.002).	FDA approval in 2021	Schmid, et al. 2020; Schmid, et al. 2022; Schmid, et al. 2024 [2,3,12]
KEYLYNK-009 (NCT04191135)	After induction pembrolizumab + platinum-based chemotherapy, patients with clinical benefit were randomized to two Arms. Arm 1: pembrolizumab (200 mg IV every 3 weeks) + olaparib (a PARP inhibitor); *n* = 135. Arm 2: pembrolizumab (same as Arm 1) + chemotherapy; *n* = 136. Chemotherapy options included carboplatin + gemcitabine.	2020	II	Locally recurrent inoperable or metastatic TNBC	Maintenance/post-induction after first-line pembrolizumab + chemotherapy	Arm 1 vs. Arm 2, all patients, regardless of PD-L1 status: Median PFS: 5.5 vs. 5.6 months (HR = 0.98, *p* = 0.4556). Median OS: 25.1 vs. 23.4 months (HR = 0.95).	/	Rugo, et al. 2026 [14]
KEYNOTE-D19/ASCENT04 (NCT05382286)	Arm 1: pembrolizumab (200 mg IV every 3 weeks, for up to 2 years) + sacituzumab govitecan (an antibody-drug conjugate); *n* = 221. Arm 2: pembrolizumab (same as Arm 1) + physician’s-choice chemotherapy; *n* = 222. Chemotherapy options included paclitaxel, nab-paclitaxel, or gemcitabine + carboplatin.	2022	III	Previously untreated PD-L1-positive locally advanced unresectable or metastatic TNBC	First-line metastatic treatment	Arm 1 vs. Arm 2 (PD-L1-positive) ORR: 60% vs. 53%. Median PFS: 11.2 vs. 7.8 months (HR = 0.65, *p* < 0.001). Median DOR: 16.5 vs. 9.2 months.	Not yet FDA-approved for TNBC	Tolaney, et al. 2026 [15]
**Anti-PD-1 antibody: nivolumab**								
TONIC (NCT02499367)	Randomized, non-comparative adaptive trial. All arms received nivolumab after 2-week induction or no induction. Arm 1: radiotherapy induction + nivolumab (3 mg/kg IV every 2 weeks); *n* = 12. Arm 2: doxorubicin induction + nivolumab (same as Arm 1); *n* = 17. Arm 3: cyclophosphamide induction + nivolumab (same as Arm 1); *n* = 12. Arm 4: cisplatin induction + nivolumab (same as Arm 1); *n* = 13. Arm 5: no induction, nivolumab alone (same as Arm 1); *n* = 12.	2015	II	Metastatic TNBC	/	All patients, regardless of PD-L1 status: ORR, overall: 20% ORR by arm: Arm 1: 8% (Arm 1), 35% (Arm 2), 8% (Arm 3), 23% (Arm 4), 17% (Arm 5). Median PFS: ~1.9 months.	/	Voorwerk, et al. 2019 [16]
OXEL (NCT03487666)	Arm 1: nivolumab (360 mg IV every 3 weeks for 6 cycles) + capecitabine; *n* = 15. Arm 2: nivolumab (same as Arm 1); *n* = 15. Arm 3: capecitabine; *n* = 14.	2018	II	Residual high-risk early-stage TNBC after neoadjuvant chemotherapy and surgery	Post-neoadjuvant treatment	All patients, regardless of PD-L1 status: PIS change from baseline to week 6: 91% (Arm 1), 47% (Arm 2), 53% (Arm 3) (*p* = 0.08 across three arms) Median iDFS: 21.37 months (Arm 1), 16.75 months (Arm 2), 16.04 months (Arm 3), (log-rank *p* = 0.0798 across three arms)	/	Lynce, et al. 2024 [17]
**Anti-PD-L1 antibody: atezolizumab**								
IMpassion130 (NCT02425891)	Arm 1: atezolizumab (840 mg IV on day 1 and 14 of each 28-day cycle) + nab-paclitaxel; *n* = 451. Arm 2: placebo + nab-paclitaxel; *n* = 451.	2015	III	Previously untreated unresectable locally advanced or metastatic TNBC	First-line advanced/metastatic treatment	Arm 1 vs. Arm 2, ITT population. regardless of PD-L1 status: ORR: 56% vs. 45.9%. Median PFS: 7.2 vs. 5.5 months (HR = 0.80, *p* = 0.0025) Median OS: 21.0 vs. 18.7 months (HR = 0.87, *p* = 0.077) Arm 1 vs. Arm 2, PD-L1-positive (IC ≥ 1%): ORR: 58.9% vs. 42.6%. Median PFS: 7.5 vs. 5.0 months (HR = 0.62, *p* < 0.0001). Median OS: 25.4 vs. 17.9 months, (HR = 0.67, *p* = 0.0016)	FDA approval in 2019.	Schmid, et al. 2018 [4]
IMpassion031 (NCT03197935)	Arm 1: atezolizumab + neoadjuvant chemotherapy, followed by adjuvant atezolizumab; *n* = 165. Arm 2: placebo + neoadjuvant chemotherapy, followed by adjuvant placebo; *n* = 168. Atezolizumab was given 840 mg IV every 2 weeks during the neoadjuvant phase and 1200 mg IV every 3 weeks during the adjuvant phase. Chemotherapy included nab-paclitaxel followed by doxorubicin + cyclophosphamide.	2017	III	Stage II–III early-stage TNBC	First-line neoadjuvant/adjuvant treatment	Arm 1 vs. Arm 2, ITT population, regardless of PD-L1 status: pCR: 58% vs. 41% (*p* = 0.0044). Arm 1 vs. Arm 2, PD-L1-positive (IC ≥ 1%): pCR: 69% vs. 49% (*p* = 0.021).	Has not received FDA approval.	Mittendorf, et al. 2020 [19]
IMpassion131 (NCT03125902)	Arm 1: atezolizumab (840 mg IV on day 1 and 14 of each 28-day cycle) + paclitaxel; *n* = 431. Arm 2: placebo + paclitaxel; *n* = 220.	2017	III	Previously untreated unresectable locally advanced or metastatic TNBC	First-line advanced/metastatic treatment	Arm 1 vs. Arm 2, ITT population, regardless of PD-L1 status: Median PFS: 5.7 vs. 5.6 months (HR = 0.86). Median OS: 19.2 vs. 22.8 months (HR = 1.12). Arm 1 vs. Arm 2, PD-L1-positive (IC ≥ 1%): Median PFS: 6.0 vs. 5.7 months (HR = 0.82, *p* = 0.20). Median OS: 22.1 vs. 28.3 months (HR = 1.11).	Contributed to withdrawal of U.S. atezolizumab TNBC indication in 2021.	Miles, et al. 2021 [5]
IMpassion030/ALEXANDRA (NCT03498716)	Arm 1: adjuvant atezolizumab + standard chemotherapy; *n* = 1101. Arm 2: standard chemotherapy alone; *n* = 1098. Atezolizumab was given 840 mg IV every 2 weeks for up to 10 doses, then 1200 mg IV every 3 weeks for up to 1 year. Chemotherapy included standard adjuvant anthracycline/taxane-based treatment.	2018	III	Stage II–III high-risk early-stage TNBC after surgery	Adjuvant treatment after surgery	Arm 1 vs. Arm 2, ITT population, regardless of PD-L1 status: 3-year iDFS: 84.6% vs. 86.4% (HR = 1.11, *p* = 0.38). 3-year OS: 92.3% vs. 93.7% (HR = 1.23).	/	Ignatiadis, et al. 2025 [20]
IMpassion132 (NCT03371017)	Arm 1: atezolizumab (1200 mg IV every 3 weeks) + physician’s-choice chemotherapy; *n* = 298. Arm 2: placebo + physician’s-choice chemotherapy; *n* = 297. Chemotherapy options included gemcitabine + carboplatin or capecitabine.	2018	III	Inoperable locally advanced or metastatic TNBC relapsing < 12 months after curative-intent therapy for early TNBC	First-line treatment for early-relapsing advanced/metastatic disease	Arm 1 vs. Arm 2, PD-L1-positive primary population (IC ≥ 1%): ORR: 40% vs. 28%. Median OS: 12.1 vs. 11.2 months (HR = 0.93, *p* = 0.59). 12-month OS rate: 50.3% vs. 47.6%.	/	Dent, et al. 2024 [18]
**Anti-CTLA4 antibody (ipilimumab) + anti-PD-L1 antibody or bispecific antibody**								
BELLINI (NCT03815890)	Cohort A: nivolumab alone for 4 weeks; *n* = 16. Cohort B: nivolumab + low-dose ipilimumab for 4 weeks; *n* = 15. Cohort C: nivolumab + low-dose ipilimumab for 6 weeks, followed by surgery; *n* = 15. Nivolumab was given 240 mg IV on days 1 and 15, and ipilimumab was given 1 mg/kg IV on day 1.	2019	II	Early-stage TNBC selected by TILs; Cohort C required high TILs, ≥ 50%, and node-negative disease.	First-line neoadjuvant treatment	Cohort C: pCR: 33.3% (5/15). Major pathological response: 53% (8/15).	/	Nederlof, et al. 2024 [21]
BREASTIMMUNE-03 (NCT03818685)	Arm 1: nivolumab + ipilimumab after surgery; *n* = 50. Nivolumab was given 360 mg IV every 3 weeks for 8 doses, and ipilimumab was given 1 mg/kg IV every 6 weeks for 4 doses. Arm 2: capecitabine after surgery; *n* = 50.	2019	II	High-risk early-stage TNBC with residual disease after neoadjuvant chemotherapy	Adjuvant treatment	Arm 1 vs. Arm 2, ITT population, regardless of PD-L1 status: DFS at 34.4 months: 38% vs. 44% (HR = 0.84, *p* = 0.5938).	/	Trédan, et al. 2026 [22]
KN046 phase II (NCT03872791)	Single arm: KN046 (an anti-PD-L1 × anti-CTLA-4 bispecific antibody, 3 mg/kg or 5 mg/kg every 2 weeks) + nab-paclitaxel; *n* = 25 evaluable.	2019	Ib/II	Locally advanced inoperable or metastatic TNBC	First-line advanced/metastatic treatment	All patients, regardless of PD-L1 status: ORR: 44%. Median PFS: 7.3 months. Median OS: 30.9 months. PD-L1-positive vs. PD-L1-negative: Median PFS: 8.6 vs. 4.7 months. 2-year OS rate: 62.5% vs. 57.1%.	/	Li, et al. 2024 [23]

ORR: objective response rate, defined as the percentage of patients with measurable tumor shrinkage, typically CR + PR by RECIST criteria. CR (complete response) indicates disappearance of all targeted lesions while PR (partial response) indicates a predefined amount of tumor shrinkage, commonly ≥ 30%. PFS: progression-free survival, defined as the time from treatment initiation or randomization to disease progression or death. OS: overall survival, defined as the time from treatment initiation or randomization to death from any cause. pCR: pathological complete response, defined as no residual invasive cancer in the resected breast and sampled lymph nodes after neoadjuvant therapy. CPS: combined positive score, a PD-L1 scoring method calculated as CPS = (PD-L1–staining tumor cells + lymphocytes + macrophages)/total viable tumor cells × 100. ITT: intention-to-treat population, including all randomized patients regardless of whether they completed treatment. HR: hazard ratio, where HR < 1 indicates a better outcome in the treatment group and HR > 1 indicates a worse outcome. DCR: disease control rate, defined as the percentage of patients with CR, PR, or stable disease. EFS: event-free survival, defined as the time from treatment initiation or randomization to recurrence, progression, second primary cancer, or death. PIS: peripheral immunoscore, an immune-response score derived from peripheral blood immune-cell features and used as an exploratory pharmacodynamic endpoint in some trials. iDFS: invasive disease-free survival, defined as the time from surgery to invasive recurrence, second primary invasive cancer, or death. IC: immune-cell score, defined as the percentage of tumor area occupied by PD-L1–positive tumor-infiltrating immune cells.

**Table 2:** Preclinical evaluation outcomes of ICI-based therapy for TNBC.

Therapy	TNBC Model	Treatment Strategy (Time, Dose, Etc.)	Surgery	Outcome (Survival Rate, Tumor Size, Etc.)	Refs.
**Syngenetic models in immunocompetent mice** **(e.g., 4T1, EMT6, EO771, TS/A and MMTV-PyMT murine cell lines inoculated in BALB/c or C57BL/6 or FVB/N mice)**					
^1^ anti-PD-1: Neoadj Adj	4T1.2, EO771 cells	2 × 10^4^ 4T1.2 or 5 × 10^4^ EO771 were inoculated into BALB/c or C57 mice (set as day 0). Treatment started at 50–100 mm^3^ (day 15–19 post-tumor inoculation). ICI: anti-PD-1 (RMP1-4, 0.1 mg/dose, i.p.). Neoadj (4T1): ICI on days 17 and 19 and surgery on day 21. Early Neoadj (4T1): ICI on days 15 and 17 and surgery on day 19. Adj (4T1): surgery on day 17 and ICI on days 21 and 23. Early Adj (4T1): surgery on day 15 and ICI on days 17 and 19. Late Adj (4T1): surgery on day 19 and ICI on days 21 and 23. Neoadj (EO771): ICI on days 14 and 16 and surgery on day 18. Adj (EO771): surgery on day 18 and ICI on days 20 and 22.	Yes	4T1.2, median survival time and survival rate at day 150: 45 days, 0% (control IgG, Neoadj) 70 days, 0% (anti-PD-1, Neoadj) 80 days, 0% (anti-PD-1, early Neoadj) 55 days, 0% (anti-PD-1, Adj) 50 days, 0% (anti-PD-1, early Adj) 53 days, 0% (anti-PD-1, late Adj) EO771, median survival time and survival rate at day 100: 50 days, 0% (control IgG, Neoadj) 55 days, 0% (anti-PD-1, Neoadj) 50 days, 0% (anti-PD-1, Adj)	Liu, et al. 2016 [52]
(1) anti-PD-1 Neoadj Adj Neoadj + Adj (2) anti-PD-1 + anti-CTLA-4 Neoadj Adj Neoadj + Adj	4T1.2 cells	2 × 10^4^ 4T1.2 were inoculated into BALB/c mice (set as day 0). Treatment started at 50–80 mm^3^ (day 12–16 post-tumor inoculation) ICI: anti-PD-1 (RMP1-4) and anti-CTLA-4 (UC10-4T10), 0.25 mg/dose, i.p. Neoadj: ICI on days 12 and 14, and surgery on day 16. Adj: surgery on day 16, and ICI on days 18, 20, 27, and 34. Neoadj + Adj: ICI on days 12, 14, 18, 20, 27, and 34, and surgery on day 16.	Yes	4T1.2, median survival time and survival rate at day 150: 60 days, 0% (anti-PD-1, Neoadj) 50 days, 0% (anti-PD-1, Adj) 80 days, 6.7% (anti-PD-1, Neoadj + Adj) 90 days, 4% (anti-PD-1 + anti-CTLA-4, Neoadj) 55 days, 0% (anti-PD-1 + anti-CTLA-4, Adj) 100 days, 12% (anti-PD-1 + anti-CTLA-4, Neoadj + Adj)	Liu, et al. 2019 [53]
anti-PD-1	EMT6 cells	5 × 10^5^ EMT6 were inoculated into BALB/c mice (set as day 0). Treatment started at 80–100 mm^3^. ICI: anti-PD-1 (RMP1-14, 10 mg/kg, i.p., given twice a week for three weeks).	No	EMT6, day 24 after tumor inoculation: ~2500 mm^3^ (control) ~500 mm^3^ (anti-PD-1)	Jin, et al. 2022 [34]
anti-PD-1	EO771, AT-3 cells	5 × 10^5^ 4T1 or AT-3 and 1 × 10^5^ mammary fibroblasts were inoculated into BALB/c or C57 mice (set as day 0). Treatment started at < 50 mm^3^ (day 3 post-tumor inoculation). ICI: anti-PD-1 (CD279, 10 mg/kg, i.p., given on days 3, 6, 9, and 12).	No	EO771, day 30 after tumor inoculation: ~350 mm^3^ (control) ~80 mm^3^ (anti-PD-1) AT-3, day 40 after tumor inoculation: ~200 mm^3^ (control) ~200 mm^3^ (anti-PD-1)	Ciavattone, et al. 2024 [35]
anti-PD-L1	4T1 cells	5 × 10^5^ 4T1 were inoculated into BALB/c mice (set as day 0). Treatment started at 50–80 mm^3^ (day 7 post-tumor inoculation). ICI: anti-PD-L1 (10F.9G2, 0.2 mg/dose, i.p., given on days 7, 10, 13, and 16).	No	4T1, day 23 after tumor inoculation: ~1100 mm^3^ (control) ~900 mm^3^ (anti-PD-L1)	Li, et al. 2023 [141]
(1) anti-PD-1 (2) anti-PD-L1	4T1 cells	5 × 10^4^ 4T1 were inoculated into BALB/c mice (set as day 0). Treatment started at 40–60 mm^2^ tumor area (day 7–10 post-tumor inoculation). ICI: anti-PD-1 (RMP1-14) or anti-PD-L1 (B7-H1). 10 mg/kg antibodies were given i.p. every other day.	No	4T1 tumor area, day 25 after tumor inoculation, median survival time, and survival rate at day 60: ~60 mm^2^, 29 days, 0% (control IgG) ~80 mm^2^, 30 days, 0% (anti-PD-1) 4T1 tumor area, day 30 after tumor inoculation, median survival time, and survival rate at day 50: ~150 mm^2^, 31 days, 0% (control) ~150 mm^2^, 31 days, 0% (anti-PD-L1)	Redmond, et al. 2023 [79]
(1) anti-CTLA-4 (2) anti-PD-1	Tumor cells from primary tumor in MMTV-PyMT	2 × 10^4^ MMTV-PyMT tumor cells were inoculated into mice. Treatment started at 100 mm^3^ (set as day 0). ICI: anti-CTLA-4 (9D9, 10 mg/kg, i.p., twice per week until endpoint). anti-PD-1 (4H2, 4 mg/kg, i.p., three times per week until endpoint).	No	PyMT, day 24 after first treatment: ~2500 mm^3^ (control) ~500 mm^3^ (anti-CTLA-4) ~800 mm^3^ (anti-PD-1)	Ekiz, et al. 2018 [36]
(1) anti-PD-1 (2) anti-CTLA-4 (3) anti-PD-1 + anti-CTLA-4	EO771 cells	1 × 10^5^ EO771 were inoculated into C57 mice (set as day 0). Treatment started at >100 mm^3^ (day 3 post-tumor inoculation). ICI: anti-PD-1 (RMP1-14) or/and anti-CTLA-4 (9D9), 0.2 mg/dose (i.p.). Both were given biweekly.	No	EO771, day 21 after tumor inoculation: ~1400 mm^3^ (anti-PD-1) ~600 mm^3^ (anti-CTLA-4) ~300 mm^3^ (anti-PD-1 + anti-CTLA-4)	Crosby, et al. 2018 [89]
(1) anti-PD-1 (2) anti-CTLA-4 (3) anti-PD-1 + anti-CTLA-4	AT-3 ova^dim^ CD73^+^ cells	5 × 10^5^ AT-3 ovadim CD73+ cells were inoculated into C57 mice. Treatment started at 20–50 mm^3^ (set as day 0). ICI: anti-PD-1 (RMP1-14, 0.2 mg/dose, i.p.) and anti-CTLA-4 (9H10, 0.15 mg/dose, i.p.). Both were given 3–4 doses with 4 days apart.	No	AT-3, on day 18 after first treatment, median survival time, and survival rate at day 100: ~70 mm^3^, 30 days, 0% (anti-PD-1) ~100 mm^3^, 30 days, 0% (anti-CTLA-4) ~20 mm^3^, 50 days, 40% (anti-PD-1 + anti-CTLA-4)	Beavis, et al. 2018 [43]
(1) anti-CTLA-4 (2) anti-PD-1 + anti-CTLA-4	4T1, EO771 cells	5 × 10^5^ 4T1 or EO771 inoculated into BALB/c or C57 mice (set as day 0). Treatment started at 50–100 mm^3^ (day 6 post-tumor inoculation). ICI: anti-CTLA-4 (9D9) and anti-PD-1 (RMP1-4), 0.2 mg/dose (i.p.). Both were given on days 6, 8, 10, and 12.	No	4T1, day 14 after tumor inoculation: ~1700 mm^3^ (control) ~800 mm^3^ (anti-CTLA-4) ~300 mm^3^ (anti-PD-1 + anti-CTLA-4) EO771, day 14 after tumor inoculation: ~400 mm^3^ (control) ~200 mm^3^ (anti-CTLA-4) ~100 mm^3^ (anti-PD-1 + anti-CTLA-4)	Bailey, et al. 2022 [37]
(1) anti-PD-L1 (2) anti-CTLA-4 (3) anti-PD-L1 + α-CTLA-4	EMT6 cells	1 × 10^5^ 4T1 or EMT6 were inoculated into BALB/c mice (set as day 0). Treatment started at 100–150 mm^3^. ICI: anti-PD-L1 (10F.9G2 and 80, 20 mg/kg, i.p.) and anti-CTLA-4 (9D9, mIgG1 or mIgG2b, 20 mg/kg, i.p.). Both were given twice a week for 4 doses.	No	EMT6 complete response rate: 71% (αPD-L1) 58% (anti-CTLA-4 mIgG1) 87.5% (anti-PD-L1 + anti-CTLA-4 mIgG1) EMT6 survival rate at day 90: 0% (control) 80% (anti-PD-L1) 55% (anti-CTLA-4 mIgG1) 90% (anti-CTLA-4 mIgG2b) 90% (anti-PD-L1 + anti-CTLA-4 mIgG1) 100% (anti-PD-L1 + anti-CTLA-4 mIgG2b)	Schofield, et al. 2021 [38]
anti-PD-L1 + anti-CTLA4	Tumor cells from primary tumor in MMTV-PyMT	5 × 10^5^ MMTV-PyMT cells were inoculated into FVB mice (set as day 0). Treatment started on day 4 post tumor inoculation. ICI: anti-PD-L1 (10F.9G2, 0.1 mg/dose, i.v.), or anti-CTLA4 (9H10, 0.1 mg/dose, i.v.). Antibodies were given on days 4, 5, and 7.	No	PyMT, day 28 after tumor inoculation, median survival time, and survival rate at day 100: ~220 mm^3^, 45 days, 0% (control) ~200 mm^3^, 60 days, 20% (anti-PD-L1 + anti-CTLA4)	Ishihara, et al. 2019 [142]
(1) anti-PD-L1 (2) anti-CTLA-4 (3) anti-PD-L1 + anti-CTLA-4	Tumor cells from primary tumor in MMTV-PyMT, EMT6 cells	1 × 10^6^ MMTV-PyMT or EMT6 cells were inoculated into FVB or BALB/c mice. Treatment started at 80–100 mm^3^ (set as day 0). ICI: anti-PD-L1 (10F.9G2), or anti-CTLA-4 (9H10), or control IgG2b. 0.2 mg/dose antibodies were given i.p. twice a week.	No	PyMT, day 14 after first treatment, median survival time, and endpoint-based event-free survival rate at day 40: ~1400 mm^3^, 18 days, 0% (control) ~800 mm^3^, 22 days, 0% (anti-PD-L1) ~1000 mm^3^, 24 days, 0% (anti-CTLA-4) ~800 mm^3^, 28 days, 0% (anti-PD-L1 + anti-CTLA-4) EMT6, day 17 after first treatment: ~2000 mm^3^ (control) ~1000 mm^3^ (anti-PD-L1 + anti-CTLA-4)	Lal, et al. 2021 [80]
anti-PD-1 or anti-PD-L1 + cyclophosphamide (CTX), doxorubicin (DOX)	4T1 cells	1 × 10^6^ 4T1 were inoculated into BALB/c mice. Treatment started at 100 mm^3^ (set as day 0). ICI: anti-PD-1 (RMP1-14, 12.5 mg/kg, i.p.) and anti-PD-L1 (10F.9G2, 12.5 mg/kg, i.p.). Both were given once weekly. Chemo: CTX (100 mg/kg, i.p.) and DOX (2 mg/kg, i.p.). Both were given once weekly.	No	4T1, day 30 after first treatment: ~900 mm^3^ (control) ~800 mm^3^ (anti-PD-1) Data unavailable (anti-PD-L1) ~500 mm^3^ (CTX + DOX) ~500 mm^3^ (CTX + DOX + anti-PD-1) ~500 mm^3^ (CTX + DOX + anti-PD-L1)	Grasselly, et al. 2018 [46]
anti-PD-L1 + vinorelbine (NVB), cyclophosphamide (CTX), 5-FU	4T1 cells	1 × 10^5^ 4T1 were inoculated into BALB/c mice (set as day 0). Treatment start time was not explicitly reported. ICI: anti-PD-1 (J43) or anti-PD-L1 (10F.9G2). 0.2 mg/dose, i.p., given every two days for a total of 5 doses. Chemo: NVB (50 mg/kg, oral, oral, daily). CTX (20 mg/kg, oral, given three times per week for three weeks). 5-FU (50 mg/kg, i.p., given three times per week for three weeks).	No	4T1, day 25 after tumor inoculation: ~900 mm^3^ (control) ~520 mm^3^ (anti-PD-1) ~400 mm^3^ (anti-PD-L1) ~150 mm^3^ (5-FU + NVB) ~100 mm^3^ (anti-PD-L1 + 5-FU + NVB) ~150 mm^3^ (NVB + CTX) ~50 mm^3^ (anti-PD-L1 + NVB + CTX)	Orecchioni, et al. 2018 [44]
^2^ anti-PD-1 + doxorubicin (DOX), taxanes (T), platinum (P), vinorelbine (NVB), cyclophosphamide (CTX)	4T1, EMT6 cells	2 × 10^5^ 4T1 or 5 × 10^5^ EMT6 were inoculated into BALB/c mice (set as day 0). Treatment started at < 50 mm^3^ (day 3 post-tumor inoculation). ICI: anti-PD-1 (RMP1-14, 0.2 mg/dose, i.p., given every two days for a total of five doses). First dose was given on day 3. Chemo: CTX (140 mg/kg, i.p.). NVB (9 mg/kg, i.p.). P (0.25 mg/kg, i.p.). DOX (2.5 mg/kg, i.p.). Chemotherapeutic drugs were given once a week for three weeks. First dose was given on day 4.	No	4T1, day 28 and day 70 after tumor inoculation: ~1250 mm^3^, NA (control) ~400 mm^3^, NA (anti-PD-1) ~200 mm^3^, NA (anti-PD-1 + DOX) ~550 mm^3^, NA (anti-PD-1 + T) ~200 mm^3^, NA (anti-PD-1 + P) ~50 mm^3^, 600 mm^3^ (CTX) ~50 mm^3^, 200 mm^3^ (anti-PD-1 + CTX) ~50 mm^3^, 200 mm^3^ (CTX + NVB) ~50 mm^3^, 150 mm^3^ (anti-PD-1 + CTX + NVB) EMT6, day 28 after tumor inoculation: ~1100 mm^3^ (control) ~300 mm^3^ (anti-PD-1) ~400 mm^3^ (anti-PD-1 + DOX) ~230 mm^3^ (anti-PD-1 + T) ~1000 mm^3^ (anti-PD-1 + P) ~200 mm^3^ (CTX) ~200 mm^3^ (anti-PD-1 + CTX) ~200 mm^3^ (CTX + NVB) ~50 mm^3^ (anti-PD-1 + CTX + NVB)	Falvo, et al. 2021 [39]
anti-PD-1 + paclitaxel (PTX)	4T1 cells	2 × 10^4^ 4T1 were inoculated into BALB/c mice (set as day 0). Treatment started at ~50 mm^3^ (day 3 post-tumor inoculation). ICI: anti-PD-1 (0.2 mg/dose, i.p., given every three days for 2 weeks). Chemo: PTX (6 mg/kg, i.p., given every 2 days for 2 weeks).	No	4T1, day 22 after tumor inoculation, median survival time, and survival rate at day 60: ~1000 mm^3^, 37 days, 0% (control) ~700 mm^3^, 42 days, 0% (anti-PD-1) ~700 mm^3^, 42 days, 0% (PTX) ~450 mm^3^, 48 days, 0% (anti-PD-1 + PTX)	Chen, et al. 2020 [47]
anti-PD-1 + paclitaxel (PTX)	EO771 cells	1 × 10^6^ EO771 were inoculated into C57 mice. Treatment started at < 20 mm^3^ (day 0 post-tumor inoculation). ICI: anti-PD-1 (RMP1-14, 0.2 mg/dose, i.p., given on days 2, 4, 6, and 8). Chemo: PTX (10 mg/kg, i.v., given on days 0, 2, 4, 6, and 8).	No	EO771, day 10 after tumor inoculation: ~900 mm^3^ (control) ~850 mm^3^ (anti-PD-1) ~550 mm^3^ (PTX) ~300 mm^3^ (anti-PD-1 + PTX)	Choi, et al. 2024 [40]
anti-PD-L1+ nintedanib (NIN), paclitaxel (PTX)	EMT6/CDDP cells	2 × 10^5^ EMT6/CDDP (cisplatin-resistant EMT-6) were inoculated into BALB/c mice (set as day 0). EMT-6/CDDP tumors were implanted, surgically resected at ~300 mm^3^, and therapy was initiated one week later when mice had established lung and draining lymph-node metastases. ICI: anti-PD-L1 (10F.9G2, 5 mg/kg, i.p., given twice a week). Chemo: NIN (50 mg/kg, oral, given daily for 2 weeks and then 5 days on and 2 days off). PTX (30 mg/kg, i.p., given once every three weeks).	Yes	EMT6/CDDP, day 27 after tumor inoculation, median survival time, and survival rate at day 70: ~1600 mm^3^, 30 days, 0% (control) ~1200 mm^3^, 31.5 days, 0% (anti-PD-L1) ~1400 mm^3^, 28 days, 0% (PTX) ~700 mm^3^, 28.5 days, 0% (anti-PD-L1 + PTX) ~550 mm^3^, 30 days, 0% (anti-PD-L1 + NIN) ~1400 mm^3^, 32.5 days, 0% (NIN) ~700 mm^3^, 27 days, 0% (PTX + NIN) ~700 mm^3^, 37 days, 0% (anti-PD-L1 + PTX + NIN)	Reguera-Nuñez, et al. 2019 [48]
anti-PD-L1 + paclitaxel (PTX)	Tumor cells from Tumor cells from primary tumor in MMTV-PyMT, EMT6 cells	1 × 10^6^ MMTV-PyMT cells or 4 × 10^5^ EMT6 cells were inoculated into FVBN/J or BALB/c mice (set as day 0). Treatment started at < 50 mm^3^ (day 5–7 after tumor inoculation). ICI: anti-PD-L1 (10F.9G2, 10 mg/kg, i.p., given twice per week). Chemo: PTX (10 mg/kg, i.p., given twice per week).	No	PyMT, day 11 after first treatment: ~550 mm^3^ (control) ~450 mm^3^ (anti-PD-L1) ~420 mm^3^ (PTX) ~350 mm^3^ (anti-PD-L1 + PTX) EMT6, day 14 after first treatment: ~350 mm^3^ (control) ~240 mm^3^ (anti-PD-L1) ~380 mm^3^ (PTX) ~250 mm^3^ (anti-PD-L1 + PTX)	Alečković, et al. 2023 [41]
anti-PD-1 + anti-CTLA4 + cisplatin (CDDP)	Tumor cells from primary tumor in MMTV-cre/Brca1^fl/fl^/p53^+/−^ mice	4 × 10^4^ tumor cells were inoculated into F1 (FVB/N × BALB/c) mice. Treatment starts at 100 mm^3^ (3 weeks after tumor inoculation, set as day 0). ICI: anti-PD-1 (0.2 mg/dose, i.p.) or anti-CTLA4 (0.15 μg/dose, i.p.). Chemo: CDDP (4 mg/kg, i.v.). Cisplatin was given on day 1 and then antibodies were given on days 2, 5, and 8. This treatment was repeated every 21 days for a total of 4 cycles.	No	MMTV-cre/Brca1^fl/fl^/p53^+/−^, median survival time and survival rate at day 120: 15 days, 0% (control) 45 days, 0% (CDDP) 16 days, 0% (anti-PD-1 + anti-CTLA-4) 40 days, 0% (anti-PD-1 + CDDP) 45 days, 0% (anti-CTLA-4 + CDDP) 80 days, 0% (anti-PD-1 + anti-CTLA-4+CDDP)	Nolan, et al. 2017 [49]
anti-PD-1 + carboplatin (CBDCA)	4T1, EMT6, EO771 cells	2 × 10^5^ 4T1 or EMT6 or 1 × 10^6^ EO771 were inoculated into BALB/c or C57 mice (set as day 0). Treatment started at ~50 mm^3^ (day 7 post-tumor inoculation). ICI: anti-PD-1 (RMP1-14, 0.1 mg/dose, i.p., given on days 7, 9, 11, 13, and 15). Chemo: CBDCA (100 mg/kg, i.p., given on days 7 and 14). Mice received neoadjuvant therapy and then surgery on day 20, followed by secondary tumor inoculation.	Yes	4T1, day 48 after tumor inoculation, and survival rate at day 150: ~1167 mm3, 0% (control) ~689 mm^3^, 0% (anti-PD-1) ~874 mm^3^, 0% (CBDCA) ~460 mm^3^, 90% (anti-PD-1 + CBDCA) EMT6, day 48 after tumor inoculation, and survival rate at day 150: ~1215 mm^3^, 0% (control) ~829 mm^3^, 0% (anti-PD-1) ~938 mm^3^, 0% (CBDCA) ~540 mm^3^, 50% (anti-PD-1 + CBDCA) EO771, day 48 after tumor inoculation, and survival rate at day 150: ~908 mm^3^, 0% (control) ~636 mm^3^, 0% (anti-PD-1) ~821 mm^3^, 0% (CBDCA) ~400 mm^3^, 70% (anti-PD-1 + CBDCA)	Gao, et al. 2020 [42]
anti-PD-L1 + cisplatin (CDDP), eribulin (Eri), anti-TGF-β	4T1, EMT6, TS/A cells	1 × 10^5^ 4T1, EMT6, or TS/A were inoculated into BALB/c mice (set as day 0). Treatment started at 50–100 mm^3^ (day 7 post-tumor inoculation). ICI: anti-PD-L1 (B7-H1, 10 mg/kg, i.p., given on days 7, 11, 14, 18, and 21). Chemo: CDDP (6 mg/kg, i.p., given on day 7), Eri (1 mg/kg, i.v., given on days 7 and 11). Others: αTGF-β (1D11.16.8, 10 mg/kg, i.p., given on days 7, 11, 14, 18, and 21).	No	4T1, median survival time, and survival rate at day 80: 33 days, 0% (control) 34 days, 0% (anti-PD-L1) 46 days, 0% (CDDP + Eri) 45 days, 0% (anti-PD-L1 + CDDP + Eri) 36 days, 0% (anti-PD-L1 + anti-TGF-β) 33 days, 0% (anti-TGF-β) 49 days, 0% (anti-TGF-β + CDDP + Eri) 62 days, 12.5% (anti-PD-L1 + anti-TGF-β + CDDP + Eri) EMT6, median survival time, and survival rate at day 120: 43 days, 0% (control) 53 days, 7.7% (CDDP + Eri) 46 days, 15.4% (anti-PD-L1 + anti-TGF-β) 120 days, 60% (anti-PD-L1 + anti-TGF-β + CDDP + Eri) TS/A, median survival time, and survival rate at day 60: 32 days, 0% (control) 32 days, 0% (CDDP + Eri) 38 days, 0% (anti-PD-L1 + anti-TGF-β) 43 days, 0% (anti-PD-L1 + anti-TGF-β + CDDP + Eri)	Kalfeist, et al. 2025 [45]
anti-PD-L1 + anti-VEGFR2	Tumor cells from primary tumor in MMTV-PyMT	1 × 10^5^ PyMT cells were inoculated into FVB/N mice. Treatment started at ~250 mm^3^ (set as day 0). ICI: anti-PD-L1 (10F.9G2, 10 mg/kg, i.p.), anti-VEGFR2 (DC101, 40 mg/kg, i.p.), control IgG2a (40 mg/kg, i.p.). Antibodies were given twice per week for two weeks.	No	PyMT, day 14 after first treatment: ~1700 mm^3^ (control IgG2a) ~1400 mm^3^ (anti-PD-1) ~1300 mm^3^ (anti-VEGFR2) ~800 mm^3^ (anti-PD-1 + VEGFR2)	Allen, et al. 2017 [143]
anti-PD-1 + anti-VEGFR2	4T1 and EMT6 cells, tumor cells from primary tumor in MMTV-PyMT	2 × 10^5^ 4T1 or 5 × 10^5^ EMT6 cells were inoculated into BALB/c mice. PyMT tumors isolated from MMTV-PyMT mice were cut into ~1 mm^3^ and inoculated into FVB mice (set as day 0). Treatment started at ~20 mm^3^. ICI and other antibodies: anti-PD-1 (RMP1-14, 10 mg/kg, i.p.), or anti-VEGFR2 (DC101, 10 or 40 mg/kg, i.p.), or control IgG2a (40 mg/kg, i.p.). Antibodies were given twice a week for two weeks.	No	4T1, tumor size on day 21 after tumor inoculation, and median survival time: ~600 mm^3^, 35 days (control IgG2a) ~500 mm^3^, 35 days (anti-PD-1) ~300 mm^3^, 40 days (40 mg/kg anti-VEGFR2) ~250 mm^3^, 50 days (anti-PD-1 + 40 mg/kg anti-VEGFR2) ~400 mm^3^, 35 days (10 mg/kg anti-VEGFR2) ~100 mm^3^, >55 days (anti-PD-1 + 10 mg/kg anti-VEGFR2) EMT6, tumor size on day 21 after tumor inoculation: ~600 mm^3^ (control IgG2a) ~580 mm^3^ (anti-PD-1) ~220 mm^3^ (10 mg/kg anti-VEGFR2) PyMT, tumor size on day 22 after tumor inoculation, and median survival time: ~900 mm^3^, 35 days (control IgG2a) ~900 mm^3^, 35 days (anti-PD-1) ~400 mm^3^, 42 days (40 mg/kg anti-VEGFR2) ~200 mm^3^, 55 days (anti-PD-1 + 40 mg/kg anti-VEGFR2) ~600 mm^3^, 35 days (10 mg/kg anti-VEGFR2) ~100 mm^3^, >60 days (anti-PD-1 + 10 mg/kg anti-VEGFR2)	Li, et al. 2020 [144]
**Patient-derived or human cell line-derived xenograft models in humanized immunocompromised mice** **(e.g., human TNBC tumor or MDA-MB-231 and MDA-MB-436 human cell lines in huNSG or other mice)**					
anti-PD-1	MDA-MB-231 cells	5 × 10^6^ MDA-MB-231 and 2.5 × 10^6^ human T cells were inoculated into PMDC-humanized NOD/SCID mice (set as day 0). Treatment started on the same day of tumor inoculation. ICI: anti-PD-1 (5G9.G1.B11, 0.5 mg/dose, i.p., given twice per week for 4 weeks).	No	MDA-MB-231 (high PD-L1 expression), day 38 after tumor inoculation, median survival time, and survival rate at day 50: ~1000 mm^3^, 35 days, 0% (control) ~1000 mm^3^, 31 days, 0% (anti-PD-1)	Chang, et al. 2017 [145]
anti-PD-1	MDA-MB-231 cells	5–10 × 10^6^ MDA-MB-231 were inoculated into 16–21-week-old CD34^+^ HSC-humanized BRGS mice. Treatment started at 150–300 mm^3^ (set as day 0). ICI: anti-PD-1 (nivolumab, i.p., given 30 mg/kg daily day 0–10, then 15 mg/kg once a week after day 10).	No	MDA-MB-231 (high PD-L1 expression), day 30 after first treatment: ~2000 mm^3^ (control) ~900 mm^3^ (anti-PD-1)	Capasso, et al. 2019 [60]
(1) anti-PD-1 (2) anti-CTLA-4	Patient-derived tumors	Patient-derived tumor samples (2 × 2 mm MC1, BCM-2147, BCM-4913, BCM-4664, BCM-5471) were transplanted in the fat pad of NSG 6–8 weeks after human CD34^+^ HSC cell injection. Treatment started at ~150 mm^3^ (set as day 0). ICI: anti-PD-1 (nivolumab, 10 mg/kg, i.v., given to mice with MC1), anti-PD-1 (pembrolizumab, 10 mg/kg, i.v., given to mice with other TNBC tumors), anti-CTLA-4 (ipilimumab, 10 mg/kg, i.v.). ICIs were given weekly for three weeks.	No	MC1 (high PD-L1 expression), day 22 after first treatment: ~800 mm^3^ (control) ~300 mm^3^ (anti-PD-1) ~780 mm^3^ (anti-CTLA-4) BCM-4913 (high PD-L1 expression), day 20 after first treatment: ~800 mm^3^ (control) ~400 mm^3^ (anti-PD-1) BCM-4664 (low PD-L1 expression), day 20 after first treatment: ~1150 mm^3^ (control) ~1200 mm^3^ (anti-PD-1) BCM-5471 (low PD-L1 expression), day 31 after first treatment: ~900 mm^3^ (control) ~900 mm^3^ (anti-PD-1)	Rosato, et al. 2018 [65]
anti-PD-1 or cisplatin (CDDP)	MDA-MB-231 cells, patient-derived tumors	5 × 10^6^ MDA-MB-231 or 20–40 uL of homogenized patient-derived tumor samples (BR1126) were transplanted to NSG 12 weeks after human CD34^+^ HSC cell injection. Treatment started at 50–120 mm^3^ (set as day 0). ICI: anti-PD-1 (pembrolizumab, 10 mg/kg on day 0, followed by 5 mg/kg on days 5, 10, 15, 20, and 25, i.p. or i.v.). Chemo: CDDP (2 mg/kg, i.v., given on days 0, 7, and 14).	No	MDA-MB-231 (high PD-L1 expression), day 26 after first treatment: ~650 mm^3^ (control) ~400 mm^3^ (anti-PD-1) BR1126 (high PD-L1 expression), day 21 after first treatment: ~320 mm^3^ (control) ~80 mm^3^ (anti-PD-1) ~20 mm^3^ (CDDP)	Wang, et al. 2018 [61]
anti-PD-1 + niraparib (Nira)	MDA-MB-436 cells	5 × 10^6^ MDA-MB-436 were inoculated into 6–8-week-old CD34^+^ HSC-humanized NOG-EXL mice. Treatment started at 90–110 mm^3^ (set as day 0). ICI: anti-PD-1 (pembrolizumab, 200 mg daily, i.p., given on days 0, 4, 9, 13, 18, 22, and 28). Chemo: Nira (35 mg/kg once daily for 5 days on and 2 days off per week for 4 weeks, oral).	No	MDA-MB-436 (high PD-L1 expression), day 28 after first treatment: ~1000 mm^3^ (control) ~550 mm^3^ (anti-PD-1) ~300 mm^3^ (Nira) ~200 mm^3^ (anti-PD-1 + Nira)	Wang, et al. 2019 [64]
(1) anti-PD-1 (2) anti-PD-L1 (3) anti-CTLA-4 (4) anti-TGFβ (5) anti-TGFβRII (6) anti-CTLA4-TGFβRII (bispecific) (7) anti-PD-L1-TGFβRII (bispecific)	MDA-MB-231 cells	2 × 10^6^ MDA-MB-231 were inoculated into CD34^+^ HSC-humanized NSG (set as day 0). Treatment started at ~100 mm^3^ (day 10 post-tumor inoculation). ICI and other antibodies: anti-PD-1 (pembrolizumab), anti-PD-L1 (atezolizumab), anti-CTLA-4 (ipilimumab), anti-TGFβ (1D11), anti-TGFβRII (α-gp120-TGFβRII). Antibodies were given at 5 mg/kg i.p. weekly.	No	MDA-MB-231 (high PD-L1 expression), day 37 after tumor inoculation: ~1300 mm^3^ (control) ~1300 mm^3^ (anti-CTLA-4) ~1200 mm^3^ (anti-TGFβ) ~1200 mm^3^ (anti-CTLA-4 + anti-PD-1) ~700 mm^3^ (anti-CTLA-4 + anti-TGFβ) ~450 mm^3^ (anti-CTLA-4-TGFβRII) ~350 mm^3^ (anti-CTLA-4-TGFβRII + anti-PD-1) MDA-MB-231, week 6 after tumor inoculation: ~580 mm^3^ (control) ~500 mm^3^ (anti-TGFβRII) ~450 mm^3^ (anti-PD-L1) ~500 mm^3^ (anti-TGFβRII + anti-PD-L1) ~140 mm^3^ (anti-PD-L1-TGFβRII)	Ravi, et al. 2018 [62]
(1) anti-PD-1 (2) anti-PD-L1 (3) anti-CD137 agonist (4) MCLA-145 (CD137×PD-L1 bispecific)	MDA-MB-231 cells	3 × 10^6^ MDA-MB-231 were inoculated into CD34^+^ HSC-humanized NSG mice (set as day 0). Treatment started at 80–100 mm^3^. ICI and other antibodies: anti-PD-1 (pembrolizumab, 5 mg/kg, i.p.), or anti-PD-L1 (acezolizumab, 5 mg/kg, i.p.), or anti-CD137 agonist (urelumab, 5 mg/kg, i.p.), or MCLA-145 (5 mg/kg, i.p.). Antibodies were given every 5 days for 31 days.	No	MDA-MB-231 (high PD-L1 expression), day 48 after tumor inoculation: ~1000 mm^3^ (control) ~750 mm^3^ (anti-PD-1) ~750 mm^3^ (anti-PD-L1) ~900 mm^3^ (anti-CD137) ~1000 mm^3^ (anti-PD-L1 + anti-CD137) ~500 mm^3^ (MCLA-145)	Geuijen, et al. 2021 [63]
**Genetically engineered mouse models** **(e.g., MMTV-PyMT)**					
anti-PD-L1	FVB/N MMTV-PyMT	Treatment starts at 6 weeks of age (set the day of birth as day 0). ICI: anti-PD-L1 (B7-H1) or control IgG. 10 mg/kg antibodies were given i.p. on days 42, 44, and 46 after birth.	No	Tumor number at week 13, median survival time, and survival rate at week 25: 5, 12 weeks, 0% (control) 5, 13 weeks, 0% (anti-PD-L1)	Redmond, et al. 2023 [79]
(1) anti-PD-L1 (2) anti-CTLA-4 (3) anti-PD-L1 + anti-CTLA-4	FVB/N MMTV-PyMT	MMTV-PyMT mice were treated when tumor size reach 80–100 mm^3^ (~80-day-old, set as day 0). ICI: anti-PD-L1 (10F.9G2), or anti-CTLA-4 (9H10), or control IgG2b. 0.2 mg/dose antibodies were given i.p. twice a week.	No	Tumor size on day 14, median survival time, and survival rate at day 40: ~1800 mm^3^, 32 days, 0% (control) ~1800 mm^3^, 30 days, 0% (anti-PD-L1) ~1800 mm^3^, 30 days, 0% (anti-CTLA-4) ~1800 mm^3^, 30 days, 0% (anti-PD-L1 + anti-CTLA-4)	Lal, et al. 2021 [80]
anti-PD-1 + doxorubicin (DOX)	C57BL/6 MMTV-PyMT	Treatment started when the first tumor reached 60 mm in diameter (set as day 0). ICI: anti-PD-1 (4H2, i.p.), control IgG (i.p.). Antibodies were given 20 mg/kg on day 0 or 1, followed by 10 mg/kg weekly for a total of 4 doses. Chemo: DOX (5 mg/kg, i.p., weekly for 5 weeks). First dose was given on day 0.	No	Tumor size at day 27 after first treatment (tumor was ~50 mm^3^ at day 0): ~400 mm^3^ (control IgG) ~200 mm^3^ (anti-PD-1) Tumor size at day 35 after first treatment (tumor was ~300 mm^3^ on day 0): ~350 mm^3^ (control IgG + DOX) ~200 mm^3^ (anti-PD-1 + DOX)	Sirait-Fischer, et al. 2020 [78]
anti-PD-L1 + cisplatin (CDDP), eribulin (Eri), anti-TGF-β	B6.FVB MMTV-PyMT	Treatment started at the appearance of the first tumor lesion (set as day 0). ICI: anti-PD-L1 (B7-H1, 10 mg/kg, i.p., given on days 0, 4, 8, 12, and 14). Chemo: CDDP (6 mg/kg, i.p., given on day 0), Eri (1 mg/kg, i.v., given on days 0 and 4). Others: anti-TGF-β (1D11.16.8, 10 mg/kg, i.p., given on days 0, 4, 8, 12, and 14).	No	Median survival time, % of mice with ≥ 5 tumor lesions at day 29, and survival rate at day 120: ~33 days, 58%, 0%, (control) ~50 days, 50%, 0% (CDDP + Eri) ~32 days, 67%, 0% (anti-PD-L1 + anti-TGF-β) ~80 days, 33%, 0% (anti-PD-L1 + anti-TGF-β + CDDP + Eri)	Kalfeist, et al. 2025 [45]

^1^ Neoadj: neoadjuvant therapy, refers to treatment given before surgery; Adj, adjuvant therapy, refers to treatment given after surgery. ^2^ Taxanes include paclitaxel (PTX), docetaxel (DTX), nab-paclitaxel (nab-PTX), cabazitaxel (CBZ). Platinum drugs include cisplatin (CDDP), carboplatin (CBDCA), and oxaliplatin (L-OHP). NA: not available. Notably, studies were identified from major literature databases and publisher platforms using terms related to breast cancer, immunotherapy, and chemoimmunotherapy, without date restrictions. Included studies reported in vivo immunotherapy or chemoimmunotherapy. Nanoparticle-based studies and other emerging strategies designed to enhance chemoimmunotherapy responses were excluded when their treatment approaches were not directly comparable with clinically used regimens.

## Figures and Tables

**Figure 1:**
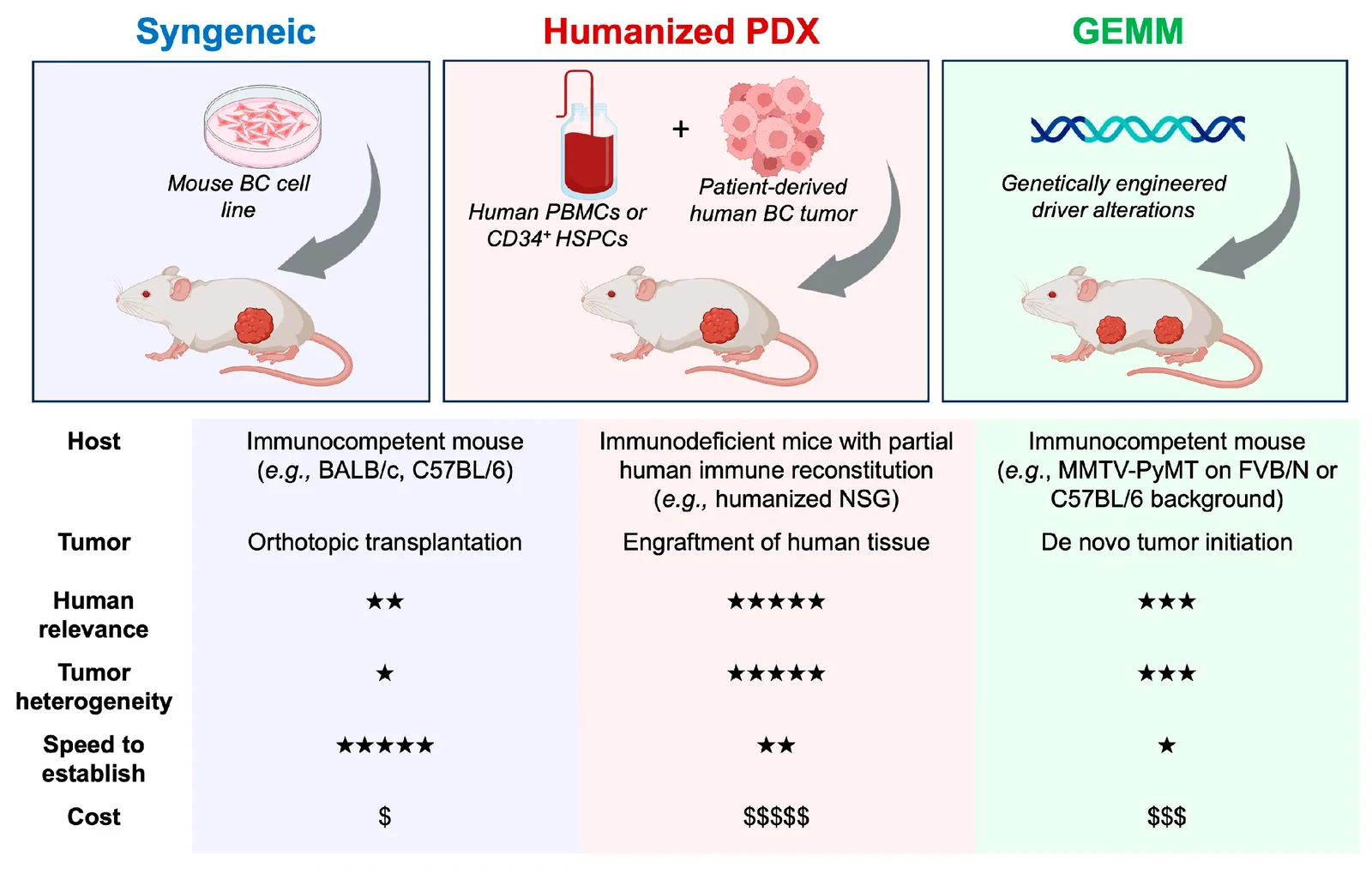
Comparison of the three major preclinical breast cancer models. Schematic overview of syngeneic, humanized patient-derived xenograft (PDX), and genetically engineered mouse model (GEMM) platforms used in breast cancer (BC) research. The figure highlights the origin of tumors, host immune status, relative human relevance, tumor heterogeneity, speed of model establishment, and associated costs for each model. Humanized PDX models are generated by engrafting patient-derived tumors into immunodeficient mice reconstituted with human peripheral blood mononuclear cells (PBMCs) or CD34^+^ hematopoietic stem cells (HSCs). Relative ratings (★) and cost ($) are qualitative and intended for comparative purposes. Created with BioRender.com.

## Data Availability

No new data were created or analyzed in this study. Data sharing is not applicable to this article.

## References

[1] Akshata Desai K.A. (2012). Triple Negative Breast Cancer—An Overview. Hered. Genet..

[2] Schmid P., Cortes J., Dent R., McArthur H., Pusztai L., Kümmel S., Denkert C., Park Y.H., Hui R., Harbeck N. (2024). Overall Survival with Pembrolizumab in Early-Stage Triple-Negative Breast Cancer. N. Engl. J. Med..

[3] Schmid P., Cortes J., Dent R., Pusztai L., McArthur H., Kümmel S., Bergh J., Denkert C., Park Y.H., Hui R. (2022). Event-free Survival with Pembrolizumab in Early Triple-Negative Breast Cancer. N. Engl. J. Med..

[4] Schmid P., Adams S., Rugo H.S., Schneeweiss A., Barrios C.H., Iwata H., Diéras V., Hegg R., Im S.-A., Shaw Wright G. (2018). Atezolizumab and Nab-Paclitaxel in Advanced Triple-Negative Breast Cancer. N. Engl. J. Med..

[5] Miles D., Gligorov J., André F., Cameron D., Schneeweiss A., Barrios C., Xu B., Wardley A., Kaen D., Andrade L. (2021). Primary results from IMpassion131, a double-blind, placebo-controlled, randomised phase III trial of first-line paclitaxel with or without atezolizumab for unresectable locally advanced/metastatic triple-negative breast cancer. Ann. Oncol..

[6] Nanda R., Chow L.Q., Dees E.C., Berger R., Gupta S., Geva R., Pusztai L., Pathiraja K., Aktan G., Cheng J.D. (2016). Pembrolizumab in Patients with Advanced Triple-Negative Breast Cancer: Phase Ib KEYNOTE-012 Study. J. Clin. Oncol..

[7] Adams S., Loi S., Toppmeyer D., Cescon D.W., De Laurentiis M., Nanda R., Winer E.P., Mukai H., Tamura K., Armstrong A. (2019). Pembrolizumab monotherapy for previously untreated, PD-L1-positive, metastatic triple-negative breast cancer: Cohort B of the phase II KEYNOTE-086 study. Ann. Oncol..

[8] Adams S., Schmid P., Rugo H.S., Winer E.P., Loirat D., Awada A., Cescon D.W., Iwata H., Campone M., Nanda R. (2019). Pembrolizumab monotherapy for previously treated metastatic triple-negative breast cancer: Cohort A of the phase II KEYNOTE-086 study. Ann. Oncol..

[9] Winer E.P., Lipatov O., Im S.A., Goncalves A., Muñoz-Couselo E., Lee K.S., Schmid P., Tamura K., Testa L., Witzel I. (2021). Pembrolizumab versus investigator-choice chemotherapy for metastatic triple-negative breast cancer (KEYNOTE-119): A randomised, open-label, phase 3 trial. Lancet Oncol..

[10] Nanda R., Liu M.C., Yau C., Shatsky R., Pusztai L., Wallace A., Chien A.J., Forero-Torres A., Ellis E., Han H. (2020). Effect of Pembrolizumab Plus Neoadjuvant Chemotherapy on Pathologic Complete Response in Women with Early-Stage Breast Cancer: An Analysis of the Ongoing Phase 2 Adaptively Randomized I-SPY2 Trial. JAMA Oncol..

[11] Schmid P., Salgado R., Park Y.H., Muñoz-Couselo E., Kim S.B., Sohn J., Im S.A., Foukakis T., Kuemmel S., Dent R. (2020). Pembrolizumab plus chemotherapy as neoadjuvant treatment of high-risk, early-stage triple-negative breast cancer: Results from the phase 1b open-label, multicohort KEYNOTE-173 study. Ann. Oncol..

[12] Schmid P., Cortes J., Pusztai L., McArthur H., Kümmel S., Bergh J., Denkert C., Park Y.H., Hui R., Harbeck N. (2020). Pembrolizumab for Early Triple-Negative Breast Cancer. N. Engl. J. Med..

[13] Cortes J., Rugo H.S., Cescon D.W., Im S.-A., Yusof M.M., Gallardo C., Lipatov O., Barrios C.H., Perez-Garcia J., Iwata H. (2022). Pembrolizumab plus Chemotherapy in Advanced Triple-Negative Breast Cancer. N. Engl. J. Med..

[14] Rugo H.S., Cescon D.W., Robson M.E., Im S.A., Dalenc F., Yañez Ruiz E., Reyes-Cosmelli F., Walshe J.M., Im Y.H., Kulyk S. (2026). KEYLYNK-009: Pembrolizumab plus Olaparib in Locally Recurrent Inoperable or Metastatic Triple-Negative Breast Cancer after Clinical Benefit from First-Line Pembrolizumab plus Chemotherapy. Clin. Cancer Res..

[15] Tolaney S.M., de Azambuja E., Kalinsky K., Loi S., Kim S.B., Yam C., Rapoport B., Im S.A., Pistilli B., McHayleh W. (2026). Sacituzumab Govitecan plus Pembrolizumab for Advanced Triple-Negative Breast Cancer. N. Engl. J. Med..

[16] Voorwerk L., Slagter M., Horlings H.M., Sikorska K., Van De Vijver K.K., De Maaker M., Nederlof I., Kluin R.J.C., Warren S., Ong S. (2019). Immune induction strategies in metastatic triple-negative breast cancer to enhance the sensitivity to PD-1 blockade: The TONIC trial. Nat. Med..

[17] Lynce F., Mainor C., Donahue R.N., Geng X., Jones G., Schlam I., Wang H., Toney N.J., Jochems C., Schlom J. (2024). Adjuvant nivolumab, capecitabine or the combination in patients with residual triple-negative breast cancer: The OXEL randomized phase II study. Nat. Commun..

[18] Dent R., André F., Gonçalves A., Martin M., Schmid P., Schütz F., Kümmel S., Swain S.M., Bilici A., Loirat D. (2024). IMpassion132 double-blind randomised phase III trial of chemotherapy with or without atezolizumab for early relapsing unresectable locally advanced or metastatic triple-negative breast cancer. Ann. Oncol..

[19] Mittendorf E.A., Zhang H., Barrios C.H., Saji S., Jung K.H., Hegg R., Koehler A., Sohn J., Iwata H., Telli M.L. (2020). Neoadjuvant atezolizumab in combination with sequential nab-paclitaxel and anthracycline-based chemotherapy versus placebo and chemotherapy in patients with early-stage triple-negative breast cancer (IMpassion031): A randomised, double-blind, phase 3 trial. Lancet.

[20] Ignatiadis M., Bailey A., McArthur H., El-Abed S., de Azambuja E., Metzger O., Chui S.Y., Dieterich M., Perretti T., Shearer-Kang E. (2025). Adjuvant Atezolizumab for Early Triple-Negative Breast Cancer: The ALEXANDRA/IMpassion030 Randomized Clinical Trial. JAMA.

[21] Nederlof I., Isaeva O.I., de Graaf M., Gielen R., Bakker N.A.M., Rolfes A.L., Garner H., Boeckx B., Traets J.J.H., Mandjes I.A.M. (2024). Neoadjuvant nivolumab or nivolumab plus ipilimumab in early-stage triple-negative breast cancer: A phase 2 adaptive trial. Nat. Med..

[22] Trédan O., Loirat D., Chabaud S., Toussaint P., Petit T., Viret F., Levy C., Robert A., Grenier J., Mansi L. (2026). Nivolumab in combination with ipilimumab versus capecitabine as post-operative treatment for triple negative breast cancer patients with residual disease after neoadjuvant chemotherapy: A multicentre, randomized, open-label phase II trial—BREASTIMMUNE-03. Breast.

[23] Li Q., Liu J., Zhang Q., Ouyang Q., Zhang Y., Liu Q., Sun T., Ye F., Zhang B., Xia S. (2024). The anti-PD-L1/CTLA-4 bispecific antibody KN046 in combination with nab-paclitaxel in first-line treatment of metastatic triple-negative breast cancer: A multicenter phase II trial. Nat. Commun..

[24] Aslakson C.J., Miller F.R. (1992). Selective events in the metastatic process defined by analysis of the sequential dissemination of subpopulations of a mouse mammary tumor. Cancer Res..

[25] Yang J., Mani S.A., Donaher J.L., Ramaswamy S., Itzykson R.A., Come C., Savagner P., Gitelman I., Richardson A., Weinberg R.A. (2004). Twist, a Master Regulator of Morphogenesis, Plays an Essential Role in Tumor Metastasis. Cell.

[26] Yip R.K.H., Rimes J.S., Capaldo B.D., Vaillant F., Mouchemore K.A., Pal B., Chen Y., Surgenor E., Murphy A.J., Anderson R.L. (2021). Mammary tumour cells remodel the bone marrow vascular microenvironment to support metastasis. Nat. Commun..

[27] Salembier R., De Haes C., Demeyere K., Van Laere S., Cervenkova L., Van Renne N., Sotiriou C., Meyer E., Steenbrugge J. (2025). Tractable mouse TNBC models capture the heterogeneous tumor immune microenvironment and adaptation to PD-L1 blockade. npj Breast Cancer.

[28] Rudqvist N.-P., Charpentier M., Lhuillier C., Wennerberg E., Spada S., Sheridan C., Zhou X.K., Zhang T., Formenti S.C., Sims J.S. (2023). Immunotherapy targeting different immune compartments in combination with radiation therapy induces regression of resistant tumors. Nat. Commun..

[29] Johnstone C.N., Smith Y.E., Cao Y., Burrows A.D., Cross R.S.N., Ling X., Redvers R.P., Doherty J.P., Eckhardt B.L., Natoli A.L. (2015). Functional and molecular characterisation of EO771.LMB tumours, a new C57BL/6-mouse-derived model of spontaneously metastatic mammary cancer. Dis. Model. Mech..

[30] Brockwell N.K., Owen K.L., Zanker D., Spurling A., Rautela J., Duivenvoorden H.M., Baschuk N., Caramia F., Loi S., Darcy P.K. (2017). Neoadjuvant Interferons: Critical for Effective PD-1–Based Immunotherapy in TNBC. Cancer Immunol. Res..

[31] Franklin D.A., James J.L., Axelrod M.L., Balko J.M. (2020). MEK inhibition activates STAT signaling to increase breast cancer immunogenicity via MHC-I expression. Cancer Drug Resist..

[32] Li R., Qi Y., Han M., Geng B., Wang G., Han M. (2021). Computed tomography reveals microenvironment changes in premetastatic lung. Eur. Radiol..

[33] Piranlioglu R., Lee E., Ouzounova M., Bollag R.J., Vinyard A.H., Arbab A.S., Marasco D., Guzel M., Cowell J.K., Thangaraju M. (2019). Primary tumor-induced immunity eradicates disseminated tumor cells in syngeneic mouse model. Nat. Commun..

[34] Jin Y., An X., Mao B., Sun R., Kumari R., Chen X., Shan Y., Zang M., Xu L., Muntel J. (2022). Different syngeneic tumors show distinctive intrinsic tumor-immunity and mechanisms of actions (MOA) of anti-PD-1 treatment. Sci. Rep..

[35] Ciavattone N.G., Guan N., Farfel A., Stauff J., Desmond T., Viglianti B.L., Scott P.J.H., Brooks A.F., Luker G.D. (2024). Evaluating immunotherapeutic outcomes in triple-negative breast cancer with a cholesterol radiotracer in mice. JCI Insight.

[36] Ekiz H.A., Lai S.-C.A., Gundlapalli H., Haroun F., Williams M.A., Welm A.L. (2018). Inhibition of RON kinase potentiates anti-CTLA-4 immunotherapy to shrink breast tumors and prevent metastatic outgrowth. OncoImmunology.

[37] Bailey C.M., Liu Y., Liu M., Du X., Devenport M., Zheng P., Liu Y., Wang Y. (2022). Targeting HIF-1α abrogates PD-L1–mediated immune evasion in tumor microenvironment but promotes tolerance in normal tissues. J. Clin. Investig..

[38] Schofield D.J., Percival-Alwyn J., Rytelewski M., Hood J., Rothstein R., Wetzel L., McGlinchey K., Adjei G., Watkins A., Machiesky L. (2021). Activity of murine surrogate antibodies for durvalumab and tremelimumab lacking effector function and the ability to deplete regulatory T cells in mouse models of cancer. mAbs.

[39] Falvo P., Orecchioni S., Hillje R., Raveane A., Mancuso P., Camisaschi C., Luzi L., Pelicci P., Bertolini F. (2021). Cyclophosphamide and Vinorelbine Activate Stem-Like CD8+ T Cells and Improve Anti-PD-1 Efficacy in Triple-Negative Breast Cancer. Cancer Res..

[40] Choi Y., Kim S.A., Jung H., Kim E., Kim Y.K., Kim S., Kim J., Lee Y., Jo M.K., Woo J. (2024). Novel insights into paclitaxel’s role on tumor-associated macrophages in enhancing PD-1 blockade in breast cancer treatment. J. Immunother. Cancer.

[41] Alečković M., Li Z., Zhou N., Qiu X., Lulseged B., Foidart P., Huang X.-Y., Garza K., Shu S., Kesten N. (2023). Combination Therapies to Improve the Efficacy of Immunotherapy in Triple-negative Breast Cancer. Mol. Cancer Ther..

[42] Gao M., Wang T., Ji L., Bai S., Tian L., Song H. (2020). Therapy with Carboplatin and Anti-PD-1 Antibodies Before Surgery Demonstrates Sustainable Anti-Tumor Effects for Secondary Cancers in Mice with Triple-Negative Breast Cancer. Front. Immunol..

[43] Beavis P.A., Henderson M.A., Giuffrida L., Davenport A.J., Petley E.V., House I.G., Lai J., Sek K., Milenkovski N., John L.B. (2018). Dual PD-1 and CTLA-4 Checkpoint Blockade Promotes Antitumor Immune Responses through CD4^+^Foxp3^−^ Cell–Mediated Modulation of CD103^+^ Dendritic Cells. Cancer Immunol. Res..

[44] Orecchioni S., Talarico G., Labanca V., Calleri A., Mancuso P., Bertolini F. (2018). Vinorelbine, cyclophosphamide and 5-FU effects on the circulating and intratumoural landscape of immune cells improve anti-PD-L1 efficacy in preclinical models of breast cancer and lymphoma. Br. J. Cancer.

[45] Kalfeist L., Ledys F., Petit S., Poirrier C., Kada Mohammed S., Galland L., Derangère V., Ilie A., Rageot D., Aucagne R. (2025). Co-targeting TGF-β and PD-L1 sensitizes triple-negative breast cancer to experimental immunogenic cisplatin-eribulin chemotherapy doublet. J. Clin. Investig..

[46] Grasselly C., Denis M., Bourguignon A., Talhi N., Mathe D., Tourette A., Serre L., Jordheim L.P., Matera E.L., Dumontet C. (2018). The Antitumor Activity of Combinations of Cytotoxic Chemotherapy and Immune Checkpoint Inhibitors Is Model-Dependent. Front. Immunol..

[47] Chen Q., Xia R., Zheng W., Zhang L., Li P., Sun X., Shi J. (2020). Metronomic paclitaxel improves the efficacy of PD-1 monoclonal antibodies in breast cancer by transforming the tumor immune microenvironment. Am. J. Transl. Res..

[48] Reguera-Nuñez E., Xu P., Chow A., Man S., Hilberg F., Kerbel R.S. (2019). Therapeutic impact of Nintedanib with paclitaxel and/or a PD-L1 antibody in preclinical models of orthotopic primary or metastatic triple negative breast cancer. J. Exp. Clin. Cancer Res..

[49] Nolan E., Savas P., Policheni A.N., Darcy P.K., Vaillant F., Mintoff C.P., Dushyanthen S., Mansour M., Pang J.-M.B., Fox S.B. (2017). Combined immune checkpoint blockade as a therapeutic strategy for *BRCA1*-mutated breast cancer. Sci. Transl. Med..

[50] Gu L., Khadaroo P.A., Su H., Kong L., Chen L., Wang X., Li X., Zhu H., Zhong X., Pan J. (2019). The safety and tolerability of combined immune checkpoint inhibitors (anti-PD-1/PD-L1 plus anti-CTLA-4): A systematic review and meta-analysis. BMC Cancer.

[51] Van Norman G.A. (2019). Limitations of Animal Studies for Predicting Toxicity in Clinical Trials: Is it Time to Rethink Our Current Approach?. JACC Basic Transl. Sci..

[52] Liu J., Blake S.J., Yong M.C.R., Harjunpää H., Ngiow S.F., Takeda K., Young A., O’Donnell J.S., Allen S., Smyth M.J. (2016). Improved Efficacy of Neoadjuvant Compared to Adjuvant Immunotherapy to Eradicate Metastatic Disease. Cancer Discov..

[53] Liu J., O’Donnell J.S., Yan J., Madore J., Allen S., Smyth M.J., Teng M.W.L. (2019). Timing of neoadjuvant immunotherapy in relation to surgery is crucial for outcome. OncoImmunology.

[54] Yip H., Haupt C., Maresh G., Zhang X., Li L. (2019). Humanized mice for immune checkpoint blockade in human solid tumors. Am. J. Clin. Exp. Urol..

[55] Cleris L., Daidone M.G., Fina E., Cappelletti V. (2019). The Detection and Morphological Analysis of Circulating Tumor and Host Cells in Breast Cancer Xenograft Models. Cells.

[56] Iorns E., Drews-Elger K., Ward T.M., Dean S., Clarke J., Berry D., Ashry D.E., Lippman M. (2012). A New Mouse Model for the Study of Human Breast Cancer Metastasis. PLoS ONE.

[57] Chen M., Pockaj B., Andreozzi M., Barrett M.T., Krishna S., Eaton S., Niu R., Anderson K.S. (2018). JAK2 and PD-L1 Amplification Enhance the Dynamic Expression of PD-L1 in Triple-negative Breast Cancer. Clin. Breast Cancer.

[58] Fricke I.B., De Souza R., Costa Ayub L., Francia G., Kerbel R., Jaffray D.A., Zheng J. (2018). Spatiotemporal assessment of spontaneous metastasis formation using multimodal in vivo imaging in HER2+ and triple negative metastatic breast cancer xenograft models in mice. PLoS ONE.

[59] Mohan N., Hosain S., Zhao J., Shen Y., Luo X., Jiang J., Endo Y., Wu W.J. (2019). Atezolizumab potentiates Tcell-mediated cytotoxicity and coordinates with FAK to suppress cell invasion and motility in PD-L1^+^ triple negative breast cancer cells. OncoImmunology.

[60] Capasso A., Lang J., Pitts T.M., Jordan K.R., Lieu C.H., Davis S.L., Diamond J.R., Kopetz S., Barbee J., Peterson J. (2019). Characterization of immune responses to anti-PD-1 mono and combination immunotherapy in hematopoietic humanized mice implanted with tumor xenografts. J. Immunother. Cancer.

[61] Wang M., Yao L.C., Cheng M., Cai D., Martinek J., Pan C.X., Shi W., Ma A.H., De Vere White R.W., Airhart S. (2018). Humanized mice in studying efficacy and mechanisms of PD-1-targeted cancer immunotherapy. FASEB J..

[62] Ravi R., Noonan K.A., Pham V., Bedi R., Zhavoronkov A., Ozerov I.V., Makarev E., Artemov V.A., Wysocki P.T., Mehra R. (2018). Bifunctional immune checkpoint-targeted antibody-ligand traps that simultaneously disable TGFβ enhance the efficacy of cancer immunotherapy. Nat. Commun..

[63] Geuijen C., Tacken P., Wang L.-C., Klooster R., Van Loo P.F., Zhou J., Mondal A., Liu Y.-B., Kramer A., Condamine T. (2021). A human CD137×PD-L1 bispecific antibody promotes anti-tumor immunity via context-dependent T cell costimulation and checkpoint blockade. Nat. Commun..

[64] Wang Z., Sun K., Xiao Y., Feng B., Mikule K., Ma X., Feng N., Vellano C.P., Federico L., Marszalek J.R. (2019). Niraparib activates interferon signaling and potentiates anti-PD-1 antibody efficacy in tumor models. Sci. Rep..

[65] Rosato R.R., Dávila-González D., Choi D.S., Qian W., Chen W., Kozielski A.J., Wong H., Dave B., Chang J.C. (2018). Evaluation of anti-PD-1-based therapy against triple-negative breast cancer patient-derived xenograft tumors engrafted in humanized mouse models. Breast Cancer Res..

[66] Choi Y., Lee S., Kim K., Kim S.-H., Chung Y.-J., Lee C. (2018). Studying cancer immunotherapy using patient-derived xenografts (PDXs) in humanized mice. Exp. Mol. Med..

[67] Chuprin J., Buettner H., Seedhom M.O., Greiner D.L., Keck J.G., Ishikawa F., Shultz L.D., Brehm M.A. (2023). Humanized mouse models for immuno-oncology research. Nat. Rev. Clin. Oncol..

[68] Kanikarla Marie P., Sorokin A.V., Bitner L.A., Aden R., Lam M., Manyam G., Woods M.N., Anderson A., Capasso A., Fowlkes N. (2022). Autologous humanized mouse models to study combination and single-agent immunotherapy for colorectal cancer patient-derived xenografts. Front. Oncol..

[69] Brehm M.A., Shultz L.D., Luban J., Greiner D.L. (2013). Overcoming current limitations in humanized mouse research. J. Infect. Dis..

[70] Fu J., Kim Y.J. (2020). Autologously Humanized Mice for Immune-Oncologic Studies. Curr. Protoc. Pharmacol..

[71] Morton J.J., Bird G., Refaeli Y., Jimeno A. (2016). Humanized Mouse Xenograft Models: Narrowing the Tumor–Microenvironment Gap. Cancer Res..

[72] Diaz-Cruz E.S., Cabrera M.C., Nakles R., Rutstein B.H., Furth P.A. (2010). BRCA1 deficient mouse models to study pathogenesis and therapy of triple negative breast cancer. Breast Dis..

[73] Raji L.M., Siddique M.M., Bohm M.S., Pierre J.F., Playdon M.C., Summers S.A., Li B., Cook K.L., Murphy E.A., Makowski L. (2025). Murine Models of Obesity-Related Cancer Risk. Cancer Prev. Res..

[74] Regua A.T., Arrigo A., Doheny D., Wong G.L., Lo H.W. (2021). Transgenic mouse models of breast cancer. Cancer Lett..

[75] Usary J., Darr D.B., Pfefferle A.D., Perou C.M. (2016). Overview of Genetically Engineered Mouse Models of Distinct Breast Cancer Subtypes. Curr. Protoc. Pharmacol..

[76] Davie S.A., Maglione J.E., Manner C.K., Young D., Cardiff R.D., Macleod C.L., Ellies L.G. (2007). Effects of FVB/NJ and C57Bl/6J strain backgrounds on mammary tumor phenotype in inducible nitric oxide synthase deficient mice. Transgenic Res..

[77] Mei Y., Wang M., Lu G., Li J., Peng L., Lang Y., Yang M., Jiang L., Li C., Zheng L. (2021). Postponing tumor onset and tumor progression can be achieved by alteration of local tumor immunity. Cancer Cell Int..

[78] Sirait-Fischer E., Olesch C., Fink A.F., Berkefeld M., Huard A., Schmid T., Takeda K., Brüne B., Weigert A. (2020). Immune Checkpoint Blockade Improves Chemotherapy in the PyMT Mammary Carcinoma Mouse Model. Front. Oncol..

[79] Redmond W.L., Kasiewicz M.J., Akporiaye E.T. (2023). Enhancement of anti-tumor efficacy of immune checkpoint blockade by alpha-TEA. Front. Immunol..

[80] Lal J.C., Townsend M.G., Mehta A.K., Oliwa M., Miller E., Sotayo A., Cheney E., Mittendorf E.A., Letai A., Guerriero J.L. (2021). Comparing syngeneic and autochthonous models of breast cancer to identify tumor immune components that correlate with response to immunotherapy in breast cancer. Breast Cancer Res..

[81] Chen X., Song X., Li K., Zhang T. (2019). FcγR-Binding Is an Important Functional Attribute for Immune Checkpoint Antibodies in Cancer Immunotherapy. Front. Immunol..

[82] Martineau R., Susini S., Marabelle A. (2024). Fc Effector Function of Immune Checkpoint Blocking Antibodies in Oncology. Immunol. Rev..

[83] Lee H.T., Lee J.Y., Lim H., Lee S.H., Moon Y.J., Pyo H.J., Ryu S.E., Shin W., Heo Y.-S. (2017). Molecular mechanism of PD-1/PD-L1 blockade via anti-PD-L1 antibodies atezolizumab and durvalumab. Sci. Rep..

[84] Simpson T.R., Li F., Montalvo-Ortiz W., Sepulveda M.A., Bergerhoff K., Arce F., Roddie C., Henry J.Y., Yagita H., Wolchok J.D. (2013). Fc-dependent depletion of tumor-infiltrating regulatory T cells co-defines the efficacy of anti-CTLA-4 therapy against melanoma. J. Exp. Med..

[85] Bu M.T., Yuan L., Klee A.N., Freeman G.J. (2022). A Comparison of Murine PD-1 and PD-L1 Monoclonal Antibodies. Monoclon. Antib. Immunodiagn. Immunother..

[86] Bruhns P. (2012). Properties of mouse and human IgG receptors and their contribution to disease models. Blood.

[87] Dahan R., Sega E., Engelhardt J., Selby M., Jeffrey A. (2015). FcγRs Modulate the Anti-tumor Activity of Antibodies Targeting the PD-1/PD-L1 Axis. Cancer Cell.

[88] Ingram J.R., Blomberg O.S., Rashidian M., Ali L., Garforth S., Fedorov E., Fedorov A.A., Bonanno J.B., Le Gall C., Crowley S. (2018). Anti–CTLA-4 therapy requires an Fc domain for efficacy. Proc. Natl. Acad. Sci. USA.

[89] Crosby E.J., Wei J., Yang X.Y., Lei G., Wang T., Liu C.-X., Agarwal P., Korman A.J., Morse M.A., Gouin K. (2018). Complimentary mechanisms of dual checkpoint blockade expand unique T-cell repertoires and activate adaptive anti-tumor immunity in triple-negative breast tumors. OncoImmunology.

[90] Wang X., Collet L., Rediti M., Debien V., De Caluwé A., Venet D., Romano E., Rothé F., Sotiriou C., Buisseret L. (2023). Predictive Biomarkers for Response to Immunotherapy in Triple Negative Breast Cancer: Promises and Challenges. J. Clin. Med..

[91] Badve S.S., Penault-Llorca F., Reis-Filho J.S., Deurloo R., Siziopikou K.P., D’Arrigo C., Viale G. (2022). Determining PD-L1 Status in Patients with Triple-Negative Breast Cancer: Lessons Learned from IMpassion130. J. Natl. Cancer Inst..

[92] Wen Z., Luo D., Wang S., Rong R., Evers B.M., Jia L., Fang Y., Daoud E.V., Yang S., Gu Z. (2024). Deep Learning-Based H-Score Quantification of Immunohistochemistry-Stained Images. Mod. Pathol..

[93] Yang W.-C., Wei M.-F., Shen Y.-C., Huang C.-S., Kuo S.-H. (2025). CDK4/6 inhibitors synergize with radiotherapy to prime the tumor microenvironment and enhance the antitumor effect of anti-PD-L1 immunotherapy in triple-negative breast cancer. J. Biomed. Sci..

[94] Volpe A., Lang C., Lim L., Man F., Kurtys E., Ashmore-Harris C., Johnson P., Skourti E., De Rosales R.T.M., Fruhwirth G.O. (2020). Spatiotemporal PET Imaging Reveals Differences in CAR-T Tumor Retention in Triple-Negative Breast Cancer Models. Mol. Ther..

[95] Chatterjee S., Lesniak W.G., Gabrielson M., Lisok A., Wharram B., Sysa-Shah P., Azad B.B., Pomper M.G., Nimmagadda S. (2016). A humanized antibody for imaging immune checkpoint ligand PD-L1 expression in tumors. Oncotarget.

[96] Blaye C., Boyer T., Peyraud F., Domblides C., Larmonier N. (2022). Beyond Immunosuppression: The Multifaceted Functions of Tumor-Promoting Myeloid Cells in Breast Cancers. Front. Immunol..

[97] Danna E.A., Sinha P., Gilbert M., Clements V.K., Pulaski B.A., Ostrand-Rosenberg S. (2004). Surgical removal of primary tumor reverses tumor-induced immunosuppression despite the presence of metastatic disease. Cancer Res..

[98] Law A.M.K., Valdes-Mora F., Gallego-Ortega D. (2020). Myeloid-Derived Suppressor Cells as a Therapeutic Target for Cancer. Cells.

[99] Bruger A.M., Dorhoi A., Esendagli G., Barczyk-Kahlert K., van der Bruggen P., Lipoldova M., Perecko T., Santibanez J., Saraiva M., Van Ginderachter J.A. (2019). How to measure the immunosuppressive activity of MDSC: Assays, problems and potential solutions. Cancer Immunol. Immunother..

[100] Egelston C.A., Guo W., Simons D.L., Ye J., Avalos C., Solomon S.T., Nwangwu M., Nelson M.S., Tan J., Bacon E.R. (2024). Organ-Specific Immune Setpoints Underlie Divergent Immune Profiles across Metastatic Sites in Breast Cancer. Cancer Immunol. Res..

[101] Kuett L., Bollhagen A., Tietscher S., Sobottka B., Eling N., Varga Z., Moch H., De Souza N., Bodenmiller B. (2025). Distant Metastases of Breast Cancer Resemble Primary Tumors in Cancer Cell Composition but Differ in Immune Cell Phenotypes. Cancer Res..

[102] Nolan E., Kang Y., Malanchi I. (2023). Mechanisms of Organ-Specific Metastasis of Breast Cancer. Cold Spring Harb. Perspect. Med..

[103] Yang Y., Yang H.H., Hu Y., Watson P.H., Liu H., Geiger T.R., Anver M.R., Haines D.C., Martin P., Green J.E. (2017). Immunocompetent mouse allograft models for development of therapies to target breast cancer metastasis. Oncotarget.

[104] Bosiljcic M., Cederberg R.A., Hamilton M.J., Lepard N.E., Harbourne B.T., Collier J.L., Halvorsen E.C., Shi R., Franks S.E., Kim A.Y. (2019). Targeting myeloid-derived suppressor cells in combination with primary mammary tumor resection reduces metastatic growth in the lungs. Breast Cancer Res..

[105] Wang J., Ocadiz-Ruiz R., Hall M.S., Bushnell G.G., Orbach S.M., Decker J.T., Raghani R.M., Zhang Y., Morris A.H., Jeruss J.S. (2023). A synthetic metastatic niche reveals antitumor neutrophils drive breast cancer metastatic dormancy in the lungs. Nat. Commun..

[106] Serrano A., Weber T., Berthelet J., El-Saafin F., Gadipally S., Charafe-Jauffret E., Ginestier C., Mariadason J.M., Oakes S.R., Britt K. (2023). Experimental and spontaneous metastasis assays can result in divergence in clonal architecture. Commun. Biol..

[107] Dafflon C., Santamaría-Martínez A., Ordóñez-Morán P. (2020). An Intrasplenic Injection Model for the Study of Cancer Stem Cell Seeding Capacity. Methods Mol. Biol..

[108] Goddard E.T., Fischer J., Schedin P. (2016). A Portal Vein Injection Model to Study Liver Metastasis of Breast Cancer. J. Vis. Exp..

[109] Wright L.E., Ottewell P.D., Rucci N., Peyruchaud O., Pagnotti G.M., Chiechi A., Buijs J.T., Sterling J.A. (2016). Murine models of breast cancer bone metastasis. BoneKEy Rep..

[110] Kersten K., de Visser K.E., van Miltenburg M.H., Jonkers J. (2017). Genetically engineered mouse models in oncology research and cancer medicine. EMBO Mol. Med..

[111] Denkert C., von Minckwitz G., Darb-Esfahani S., Lederer B., Heppner B.I., Weber K.E., Budczies J., Huober J., Klauschen F., Furlanetto J. (2018). Tumour-infiltrating lymphocytes and prognosis in different subtypes of breast cancer: A pooled analysis of 3771 patients treated with neoadjuvant therapy. Lancet Oncol..

[112] Loi S., Drubay D., Adams S., Pruneri G., Francis P.A., Lacroix-Triki M., Joensuu H., Dieci M.V., Badve S., Demaria S. (2019). Tumor-Infiltrating Lymphocytes and Prognosis: A Pooled Individual Patient Analysis of Early-Stage Triple-Negative Breast Cancers. J. Clin. Oncol..

[113] Lehmann B.D., Jovanović B., Chen X., Estrada M.V., Johnson K.N., Shyr Y., Moses H.L., Sanders M.E., Pietenpol J.A. (2016). Refinement of Triple-Negative Breast Cancer Molecular Subtypes: Implications for Neoadjuvant Chemotherapy Selection. PLoS ONE.

[114] Alizadeh D., Trad M., Hanke N.T., Larmonier C.B., Janikashvili N., Bonnotte B., Katsanis E., Larmonier N. (2014). Doxorubicin eliminates myeloid-derived suppressor cells and enhances the efficacy of adoptive T-cell transfer in breast cancer. Cancer Res..

[115] Danilin S., Merkel A.R., Johnson J.R., Johnson R.W., Edwards J.R., Sterling J.A. (2012). Myeloid-derived suppressor cells expand during breast cancer progression and promote tumor-induced bone destruction. Oncoimmunology.

[116] Hanna A., Sun X., Sheng Q., Gonzalez-Ericsson P.I., Wescott E.C., Taylor B.C., Marshall J.L., Opalenik S.R., Toren A.L., Sanchez V. (2025). Monitoring systemic immune responses to checkpoint inhibition in breast cancer reveals host responses and mechanisms of resistance. J. Immunother. Cancer.

[117] Katuwal N.B., Park N., Pandey K., Kang M.S., Hong S.D., Ghosh M., Kim S.G., Cho Y.B., Hur J., Kim S.K. (2023). Preclinical Platform Using a Triple-negative Breast Cancer Syngeneic Murine Model to Evaluate Immune Checkpoint Inhibitors. Anticancer Res..

[118] Cheng W., Xiao X., Liao Y., Cao Q., Wang C., Li X., Jia Y. (2022). Conducive target range of breast cancer: Hypoxic tumor microenvironment. Front. Oncol..

[119] Naik A., Decock J. (2020). Lactate Metabolism and Immune Modulation in Breast Cancer: A Focused Review on Triple Negative Breast Tumors. Front. Oncol..

[120] Ojaroodi A.F., Shokravi S., Eskandarzadeh S., Ghahremanzadeh A., Alizadeh F., Amirikar F., Mobayen G., Mahrooghi D., Lahouty M., Shamsasenjan K. (2026). The hypoxic tumor microenvironment: Functional and metabolic reprogramming of key immune populations. Pathol. Res. Pract..

[121] Qian X., Zhang Q., Shao N., Shan Z., Cheang T., Zhang Z., Su Q., Wang S., Lin Y. (2019). Respiratory hyperoxia reverses immunosuppression by regulating myeloid-derived suppressor cells and PD-L1 expression in a triple-negative breast cancer mouse model. Am. J. Cancer Res..

[122] Clay R., Li K., Jin L. (2025). Metabolic Signaling in the Tumor Microenvironment. Cancers.

[123] De Martino M., Rathmell J.C., Galluzzi L., Vanpouille-Box C. (2024). Cancer cell metabolism and antitumour immunity. Nat. Rev. Immunol..

[124] Courau T., Jaszczak R.G., Samad B., Flynn E., Chew N.W., Reeder G.C., Tsui J., Teklu S., Pass L.F., Edwards A.W. (2026). Differential assembly of mouse and human tumor microenvironments. Nat. Immunol..

[125] Mestas J., Hughes C.C. (2004). Of mice and not men: Differences between mouse and human immunology. J. Immunol..

[126] Exposito F., Connolly K.A., Tang T., Chiorazzi M., Hunt B.G., Cardenas J.J., Nguyen D.X., Joshi N.S., Politi K. (2025). Preclinical Models of Solid Cancers for Testing Cancer Immunotherapies. Annu. Rev. Cancer Biol..

[127] Wege A.K. (2018). Humanized Mouse Models for the Preclinical Assessment of Cancer Immunotherapy. BioDrugs.

[128] Aouad P., Halabi K., Hayar B., Sflomos G., Brisken C., Metcalfe C., Darwiche N. (2026). Preclinical models of breast cancer metastasis: Strengths, limitations, and clinical relevance. npj Breast Cancer.

[129] Fenton M., Yoneyama M., Wennerberg E., Lund T., Tutt A., Melcher A., Demaria S., Somaiah N. (2025). The untapped potential of radiation and immunotherapy for hormone receptor-positive breast cancer. npj Breast Cancer.

[130] Lambouras M., Roelofs C., Pereira M., Gruber E., Vieusseux J.L., Lanteri P., Johnstone C.N., Muntz F., O’Toole S., Ooms L.M. (2023). Functional and Phenotypic Characterisations of Common Syngeneic Tumour Cell Lines as Estrogen Receptor-Positive Breast Cancer Models. Int. J. Mol. Sci..

[131] Lu Y., Lee B.P., Eli A.V., Lynch S.E., Faisal A.R.M., Moye J., Sorace A.G. (2026). Characterization of HER2-Positive Murine Breast Cancer Models for Investigating HER2-Targeted Therapy and Immunotherapy. Cancers.

[132] Wilson B.E., Gorrini C., Cescon D.W. (2022). Breast cancer immune microenvironment: From pre-clinical models to clinical therapies. Breast Cancer Res. Treat..

[133] (2026). Mapping where mouse models match—and miss—human tumor immunity. Nat. Immunol..

[134] Ortiz M.M.O., Andrechek E.R. (2023). Molecular Characterization and Landscape of Breast cancer Models from a multi-omics Perspective. J. Mammary Gland Biol. Neoplasia.

[135] Stylianakis D., Benjamin H.A., Martella S., De Silva P., Stylianakis I., Denaro N., Leoni V.P., Atzori L., Scartozzi M., Lambertini M. (2026). Spatial multiomics of the breast tumour microenvironment: State of the field, advancements and clinical implementation promises. Crit. Rev. Oncol. Hematol..

[136] Swiatnicki M.R., Andrechek E.R. (2019). How to Choose a Mouse Model of Breast Cancer, a Genomic Perspective. J. Mammary Gland Biol. Neoplasia.

[137] Barjij I., Lamsyah O., Kdadri S., Lkhoyaali S., Najem S., Naciri S., Inrhaouen H., Elghissassi I., Boutayeb S., Mrabti H. (2026). Spatial and temporal intratumoral heterogeneity in breast cancer: A systematic and conceptual review of single-cell and spatial omics studies. BMC Cancer.

[138] Tolaney S.M., Kalinsky K., Kaklamani V.G., D’Adamo D.R., Aktan G., Tsai M.L., O’Regan R.M., Kaufman P.A., Wilks S.T., Andreopoulou E. (2021). Eribulin Plus Pembrolizumab in Patients with Metastatic Triple-Negative Breast Cancer (ENHANCE 1): A Phase Ib/II Study. Clin. Cancer Res..

[139] Vinayak S., Tolaney S.M., Schwartzberg L., Mita M., McCann G., Tan A.R., Wahner-Hendrickson A.E., Forero A., Anders C., Wulf G.M. (2019). Open-label Clinical Trial of Niraparib Combined with Pembrolizumab for Treatment of Advanced or Metastatic Triple-Negative Breast Cancer. JAMA Oncol..

[140] Stopeck A.T., Abu-Khalaf M., Borges V., Chmielowski B., Rao R., Xie B., Dudek A.Z., Mina L., O’Shaughnessy J., Chisamore M. (2025). Phase 2 trial of imprime and pembrolizumab immunotherapy in metastatic triple negative breast cancer patients who have progressed beyond first line chemotherapy. J. Immunol..

[141] Li X., Luo X., Hu S. (2023). Modulating the tumor microenvironment improves antitumor effect of anti-PD-L1 mAb in breast cancer. BioImpacts.

[142] Ishihara J., Ishihara A., Sasaki K., Lee S.S.-Y., Williford J.-M., Yasui M., Abe H., Potin L., Hosseinchi P., Fukunaga K. (2019). Targeted antibody and cytokine cancer immunotherapies through collagen affinity. Sci. Transl. Med..

[143] Allen E., Jabouille A., Rivera L.B., Lodewijckx I., Missiaen R., Steri V., Feyen K., Tawney J., Hanahan D., Michael I.P. (2017). Combined antiangiogenic and anti-PD-L1 therapy stimulates tumor immunity through HEV formation. Sci. Transl. Med..

[144] Li Q., Wang Y., Jia W., Deng H., Li G., Deng W., Chen J., Kim B.Y.S., Jiang W., Liu Q. (2020). Low-Dose Anti-Angiogenic Therapy Sensitizes Breast Cancer to PD-1 Blockade. Clin. Cancer Res..

[145] Chang C.H., Wang Y., Li R., Rossi D.L., Liu D., Rossi E.A., Cardillo T.M., Goldenberg D.M. (2017). Combination Therapy with Bispecific Antibodies and PD-1 Blockade Enhances the Antitumor Potency of T Cells. Cancer Res..

